# The OECD Program to Validate the Rat Hershberger Bioassay to Screen Compounds for *in Vivo* Androgen and Antiandrogen Responses. Phase 1: Use of a Potent Agonist and a Potent Antagonist to Test the Standardized Protocol

**DOI:** 10.1289/ehp.8751

**Published:** 2006-02-27

**Authors:** William Owens, Errol Zeiger, Michael Walker, John Ashby, Lesley Onyon, L. Earl Gray

**Affiliations:** 1 Procter & Gamble, Cincinnati, Ohio, USA; 2 Errol Zeiger Consulting, Chapel Hill, North Carolina, USA; 3 Health Canada, Ottawa, Canada; 4 Syngenta Central Toxicology Laboratory, Macclesfield, Cheshire, United Kingdom; 5 World Health Organization, Geneva, Switzerland; 6 U.S. Environmental Protection Agency, Research Triangle Park, North Carolina, USA

**Keywords:** androgen, antiandrogen, bulbocavernosus, Cowper’s glands, endocrine disruption, flutamide, glans penis, Hershberger, levator ani, seminal vesicles, testosterone propionate, validation, ventral prostate

## Abstract

The Organisation for Economic Cooperation and Development (OECD) has completed phase 1 of the Hershberger validation intended to identify *in vivo* activity of suspected androgens and anti-androgens. Seventeen laboratories from 7 countries participated in phase 1, and results were collated and evaluated by the OECD with the support of an international committee of experts. Five androgen-responsive tissues (ventral prostate, paired seminal vesicles and coagulating glands, levator ani and bulbocavernosus muscles, glans penis, and paired Cowper’s or bulbourethral glands) were evaluated. The standardized protocols used selected doses of a reference androgen, testosterone propionate (TP), and an antiandrogen, flutamide (FLU). All laboratories successfully detected TP-stimulated increases in androgen-responsive tissue weight and decreases in TP-stimulated tissue weights when FLU was co-administered. The standardized protocols performed well under a variety of conditions (e.g., strain, diet, housing protocol, bedding). There was good agreement among laboratories with regard to the TP doses inducing significant increases in tissue weights and the FLU doses decreasing TP-stimulated tissue weights. Several additional procedures (e.g., weighing of the dorsolateral prostate and fixation of tissues before weighing) and serum component measurements (e.g., luteinizing hormone) were also included by some laboratories to assess their potential utility. The results indicated that the OECD Hershberger protocol was robust, reproducible, and transferable across laboratories. Based on this phase 1 validation study, the protocols have been refined, and the next phase of the OECD validation program will test the protocol with selected doses of weak androgen agonists, androgen antagonists, a 5α-reductase inhibitor, and chemicals having no androgenic activity.

To address the possibility that chemicals could threaten the health of humans and wildlife through their interaction with the endocrine system, the Organisation for Economic Cooperation and Development (OECD) initiated a high-priority activity in 1997 to develop guidelines for the testing of potential endocrine-active chemicals (OECD 1998). This OECD activity is managed by the Task Force on Endocrine Disrupters Testing and Assessment as part of the OECD Test Guidelines Programme and was coordinated by a Validation Management Group (VMG). The VMG included experts from eight OECD member countries with expertise in toxicology, test development and validation, endocrinology, regulatory toxicology, and biostatistics.

This report focuses on the OECD Hershberger validation program as an *in vivo* androgenic screen [i.e., androgen receptor agonists, androgen receptor antagonists, and 5α-reductase inhibitors preventing testosterone (T) conversion to the more potent dihydrotestosterone]. This fulfills, in part, the priorities to develop and validate screens and tests for estrogen, androgen, and thyroid modes of action [OECD 1998; U.S. Environmental Protection Agency (U.S. EPA) 1998] by providing a screen for androgens and anti-androgens. The advantages of the Hershberger bioassay are several: the tissues are the natural targets for androgens, the tissue growth response is relatively rapid, the tissue weights are quantitative, and no specialized facilities or equipment is necessary.

The need for an androgenic screen is based on the necessity for androgens in the *in utero* development of the male reproductive tract (Jost 1947, 1953). Antiandrogens and inhibitors of androgen synthesis have been known for more than 40 years to elicit frank male reproductive tract malformations (Bloch et al. 1971; Goldman 1971; Neumann et al. 1966, 1970). In the Hershberger bioassay, if the endogenous androgen is removed by castration, then an exogenous androgen source is needed for target tissues to grow and gain weight. Chemicals that act as agonists may be identified if they cause statistically significant increases in the weights of the target androgen-dependent tissues. Alternatively, chemicals may be identified as antagonists if they cause statistically significant decreases in the stimulated target tissue weights increase when the chemicals are co-administered with a potent androgen such as testosterone propionate (TP).

The surgically castrated male rat assay for androgens has existed in various forms for more than 70 years (Korenchevsky 1932; Korenchevsky et al. 1932). This original work used tissues such as the ventral prostate (VP), seminal vesicles and coagulating glands (SVCG), and glans penis (GP). Other investigations employed other tissues such as the male preputial glands (David et al. 1934). The assay was later modified to assess the related myotrophic action by measuring the levator ani and bulbocavernosus muscles (LABC) (Eisenberg and Gordan 1950; Eisenberg et al. 1949; Hershberger et al. 1953). Then, the assay was adapted for androgen antagonists such as flutamide (FLU) (Peets et al. 1973) by measuring interference with the action of a co-administered reference androgen. More recently, the assay has been demonstrated with weaker antagonists, such as *p*,*p*′-DDE (*p*,*p*′-dichlorodiphenyldichloroethylene; O’Connor et al. 1999) and linuron (Lambright et al. 2000).

Protocols for the pharmaceutical industry (e.g., Dorfman 1969a, 1969b) and a regulatory screen for steroidal androgens (Hilgar and Vollmer 1964) have been published. More recently, Ashby and Lefevre (2000), Yamada et al. (2000), and Yamasaki et al. (2001) have investigated protocol variables with weak antiandrogens. However, no internationally standardized protocol is available, and there has been no clear consensus on which male reproductive tract tissues to include.

The objective of the OECD Hershberger assay program is to develop a new, validated test guideline. The validation of the Hershberger assay was designed to be carried out in phases. Phase 1, reported here, was designed to test, refine, and standardize the Hershberger assay using high-potency reference agonist and antagonist; to provide data on intra- and inter-laboratory variability; and to assess the feasibility and utility of several other proposed end points and procedures.

## Design of Phase 1

The objectives of the first phase of the OECD validation of the Hershberger bioassay were to *a*) demonstrate the reproducibility and sensitivity of the responses of five male sex accessory tissues to the action of a reference androgen, TP, hereafter phase 1A; *b*) assess the utility, reproducibility, and sensitivity of various other measured end points within and among participating laboratories in response to the action of TP; *c*) study the interaction of reference TP doses with a reference androgen antagonist, FLU, on the sex accessory tissues and other end points, hereafter phase 1B; and *d*) select standard reference doses of TP and FLU for future studies with weakly potent agonists, antagonists, and nonandrogenic test substances.

### Standardized protocol

The VMG and other experts drafted a set of standardized protocol conditions for phase 1A. Those protocol conditions are provided in Supplemental Material (http://www.ehponline.org/docs/2006/8751/suppl.pdf). After phase 1A, early castration [before postnatal day (PND) 40] was found to prevent complete preputial separation that complicated the GP dissection. For phase 1B, castration was delayed to approximately PND 42. This standardized protocol called for the humane treatment of the animals and for the alleviation of suffering under OECD guidelines. Draft laboratory protocols, final laboratory protocols, and final laboratory reports were reviewed for compliance with the standardized protocol and any deviations.

Other parameters such as housing, bedding, and diet were not specified and were left to the preferences of the participating laboratories. As the Hershberger bioassay is intended to be a rapid screen for a potentially large number of chemicals, too rigorous and detailed standardization would likely constrain or even prevent practice in many of the OECD member countries.

### Dissection guidance and training

The lead laboratory (the U.S. EPA laboratory of L.E.G.), with input from other experts, developed a guidance manual to standardize the dissection procedures for the male sex accessory tissues of interest. The original manual included literature references and figures as well as sets of detailed color photographs illustrating the step-by-step dissection of each tissue [Supplemental Material (http://www.ehponline.org/docs/2006/8751/suppl.pdf)]. Two training sessions were provided for the pro-sectors from the participating laboratories: one session was held at the lead laboratory, and the other at laboratory 1 (the laboratories were assigned random code numbers, which do not follow their names or countries of origin).

### Participating laboratories

A total of 17 laboratories from France, Germany, Denmark, Japan, Korea, the United Kingdom, and the United States participated in phase 1. All laboratories participated on a voluntary and self-supporting basis and included laboratories with experience conducting the assay and others without experience before this study. Table 1 describes the laboratory measurements, and other laboratory conditions are described in Supplemental Material (http://www.ehponline.org/docs/2006/8751/suppl.pdf). In-life testing for phase 1A (all laboratories) occurred during the period June 2000 through January 2001; the in-life portion of phase 1B (seven laboratories) was between March 2001 and June 2001.

### Chemicals and selected doses

The reference androgen agonist (TP; CASRN 57-82-5) was from Sigma-Aldrich (St. Louis, MO, USA; 99.9% pure), and the reference androgen antagonist (FLU) (CASRN 13311-84-7) was from Salutas Pharma (Barleben, Germany; 99.9% pure). Single chemical lots were purchased by a central chemical repository at TNO (Netherlands Organisation for Applied Scientific Research, Zeist, the Netherlands), which distributed the chemicals to all laboratories.

The doses of TP and FLU were specified in order to assess test reproducibility among the laboratories. The selected doses for phase 1A were 0.1, 0.2, 0.4, 0.8, and 1.6 mg TP/kg-body weight (bw)/day. In phase 1B, two doses of TP were selected. To approximate the dose effective in 70% of subjects (ED_70_) for the LABC and so that the VP, SVCG, and Cowper’s glands (COWS) would have high absolute and relative responses without approaching their maximums, 0.4 mg TP/kg-bw/day was selected. The lower concentration of 0.2 mg TP/kg-bw/day was selected as the second dose. The selected FLU dose series was 0.1, 0.3, 1.0, 3.0, and 10.0 mg/kg-bw/day. All doses were prepared in corn oil, and the volumes to be administered were calculated based upon the daily body weights so as to maintain the selected doses. In all cases, the doses were to be administered for 10 consecutive days at approximately 24-hr intervals.

### Data reporting

Each participating laboratory received a standardized Excel spreadsheet format for recording the data in a consistent format and e-mail transmission to the OECD Secretariat, lead laboratory, and other statisticians. The spreadsheet recorded the laboratory personnel, parameters such as diet and rat strain as well as their suppliers and lots, protocol variables such as the dates of castration and the initiation of treatment, caging practices, and bedding. The spreadsheet contained individual worksheets to record the randomization procedures used to assign the animals to dosage groups; to record the individual animal numbers, daily body weights, times of administration, administration volumes, and any clinical signs or observations for each dosage group; and to record all mandatory and optional end points measured, for example, group and individual animal identification, dates of necropsy, entry of preputial separation observations. In addition to rapid transmission, the worksheets also provided the means to quickly calculate basic means, standard deviations, and coefficients of variation (CVs) to assist data audits. This proved essential for a rapid assessment of possible entry errors or identification of possible issues, for example, unusually large standard deviations for a group, by the OECD Secretariat and the lead laboratory. In addition, the organization and format of the data in the worksheets allowed rapid calculation of basic means, standard deviations, and CVs to assist data audits and data extraction into statistical programs.

### Statistical analyses

The lead laboratory calculated means, standard errors, and the CVs for each end point using PROC MEANS on SAS (version 6.08; SAS Institute Inc., Cary, NC, USA). Analyses of variances (ANOVAs) for each laboratory were done using PROC GLM, and then the laboratories were pooled for each test substance. Data for each end point also were analyzed as a two-way ANOVA, with dose and laboratory as main effects, so that the magnitudes of the overall dose and laboratory effects, and their interactions could be determined. Because the CV for each androgen-dependent organ weight was fairly constant as the means increased, the standard deviations being proportional to the mean, the data were log transformed. Analyses were also conducted with body weight as a covariate because this adjusts the analysis for experimental variation from several sources, such as *a*) differences in the size of the rats from laboratory to laboratory, a large component of which appeared to arise from the use of different strains, and *b*) differences in the sizes of the rats on study within a laboratory. *R*^2^ values for different effects were calculated to provide an indication of the strength of the association for an effect with an end point. Thus, the robustness of the dose response across end points, the variation from laboratory to laboratory, and to what degree the dose responses vary among laboratories, as indicated by the *R*^2^ for the laboratory by dose interaction, were analyzed. The analyses of the lead laboratory were confirmed by a separate, independent statistician.

The Secretariat also conducted additional statistical analyses of the data for the mandatory end points. Because the lead laboratory procedures are based upon a pairwise *t*-test comparison of several individual groups with the single vehicle (phase 1A) or TP dose only (phase 1B) control, Dunnett’s multiple comparison procedure for multiple pairwise comparisons was employed (Dunnett 1955, 1964; Hsu 1996), using S-Plus (version 6.1; Insightful Corp., Seattle, WA, USA). The same estimate of pooled variance is used in both tests, but in Dunnett’s test a different critical value is used to account for multiple comparisons. Due to the potential of treatment related effects on the body weight, analyses were performed with both the starting and terminal body weights. Further, because the number of groups influences the error term in the Dunnett’s approach, for the TP dose–response (phase 1A) untreated vehicle controls, if performed, were excluded from the Dunnett’s analyses, and for the FLU dose response (phase 1B), the vehicle control group, if performed, was excluded from the Dunnett’s analyses. In addition, because the same Dunnett’s approach with body weight as a covariable was used for the uterotrophic bioassay validation (Kanno et al. 2001, 2003), it was judged that similar statistical analyses should be available for both the Hershberger and the uterotrophic validation programs.

The primary difference between the two statistical approaches in practice is that the analysis of covariance *F*-test followed by a *t*-test comparison is slightly more liberal in achieving statistical significance. That is, single pairwise comparisons may achieve statistical significance in some marginal cases where Dunnett’s multiple comparisons do not. The results of both analyses are reported here side by side in the tables for the mandatory end points. Outliers were observed in a few data sets (defined as Studentized residuals > 4 or < −4), but these outliers were included in all of the statistical analyses results shown here.

Because only a log transformation was used in the original analysis, the independent analysis investigated the use of untransformed data or a square-root transformation procedure. A normality test (Wilk-Shapiro) was applied to assess whether the transformation satisfied these model assumptions. The best transformation was judged to be the one that gave the largest (nonsignificant) *p*-value for the normality test statistic.

Results were also compared using benchmark dose (BMD) methodology. In this case the BMD was defined as the dose at which the mean response is increased by two standard deviations over the mean response of the control group. This definition of the BMD allows for better comparisons among end points with inherently different variability. The Hill model [*Y* (dose) = intercept + *v* × dose*^n^*/(*k^n^* + dose*^n^*), where the intercept, *v*, *k*, and *n* are parameters to be estimated] was fit to the data using the U.S. EPA BMD software BMDS (version 1.3.1; U.S. EPA 2001). In cases where the Hill model did not converge, the high-dose group was iteratively dropped until convergence was obtained. This method was used because it was determined that most of the convergence problems were due to a flat dose–response relationship in the high-dose region. The BMD calculations were made using both the log-transformed data and the transformation determined to be most appropriate.

## Results of Phase 1A: TP Dose Response

All laboratories provided the Excel spread sheets containing all of their individual animal results and summaries of the protocol conditions used. One laboratory inadvertently administered TP in μg/kg-bw/day rather than in mg/kg-bw/day doses, and no TP effect was observed in any of the tissue weights. As a result, the data submitted by this laboratory have been omitted and are not included in the data analyses or summary tables. The laboratory means and standard deviations for each tissue for phase 1A are provided in Supplemental Material (http://www.ehponline.org/docs/2006/8751/suppl.pdf).

### VP results

**Figure 1:**
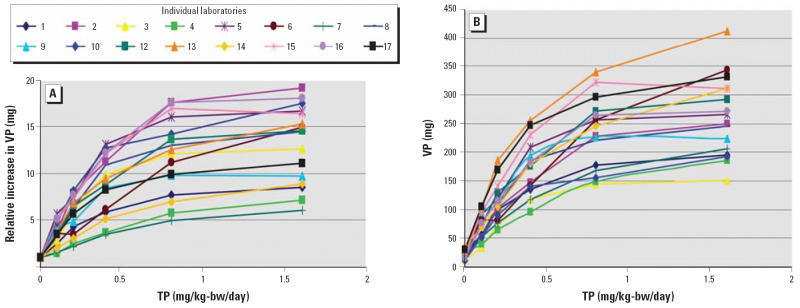
Response of VP weights to doses of TP (mg/kg-bw/day) in phase 1A. (*A*) Relative (fold) increases in the VP weights in all participating laboratories in surgically castrated male rats with TP dosing (subcutaneous) for 10 consecutive days and necropsy on day 11. (*B*) Absolute increases in the VP weights in all participating laboratories in surgically castrated male rats with TP dosing (subcutaneous) for 10 consecutive days and necropsy on day 11.

There were statistically signifi-cant, dose-dependent increases in VP weights in all laboratories. Even at the lowest dose of 0.1 mg TP/kg-bw/day, the VP weights differed significantly from the controls, except in laboratory 7 by both statistical approaches and in laboratory 4 only by the Dunnett’s test. The strain and weights of the animals at the time of initiation of dosing did not affect their ability to detect TP-induced changes in VP weight. In most laboratories, no relationships were found that resulted from differences in starting body weights and VP weight, and the laboratory-to-laboratory variability in VP weights was relatively small (*R*^2^ = 6.6%). In the pooled analysis, all TP doses led to a significant increase in VP weight. The phase 1A VP results are presented graphically both as absolute weights and relative to the vehicle control in Figure 1.

### SVCG results

There were dose-dependent increases in the SVCG weights in all laboratories. Even at the lowest dose of 0.1 mg TP/kg-bw/day, the SVCG weights differed significantly from the controls except in laboratory 7 when analyzed using both statistical approaches, and the laboratory-to-laboratory variability in SVCG weights was relatively small (*R*^2^ = 6.2%). For the SVCG, differences among the starting body weights of the animals contributed to 54% of the interlaboratory variability. However, these differences and differences in animal strains did not affect the ability of the SVCG to respond to TP. In the pooled analysis, all TP doses led to significant increases in SVCG weights.

### LABC results

There were dose-dependent increases in the LABC weights in all laboratories. At the lowest dose of 0.1 mg/kg-bw/day, the LABC was statistically significant in all laboratoies using the *t*-test approach and in almost all laboratories using the Dunnett’s approach. In laboratory 2, the LABC was marginally statistically significant by Dunnett’s approach using the starting body weights as the covariate, but not with the terminal body weights. In laboratory 9, the LABC was not significant using the Dunnett’s approach. As noted below, TP administration tended to increase body weights.

There was a deviation from the protocol in four laboratories. In these laboratories, only the levator ani muscle, and not the bulbocavernosus muscle, was dissected. This protocol deviation did not affect the laboratories’ abilities to detect weight increases in response to TP but was a source of a significant laboratory-to-laboratory variability when all laboratories were pooled (*R*^2^ = 36%). In the pooled analysis, all TP doses led to a significant increase in LABC weights regardless of the dissection procedure used.

### GP results

There were dose-dependent increases in the GP weights in all laboratories, but the absolute and relative effects were smaller than for the other androgen-dependent tissues examined. Despite this, the GP weights were statistically different from the controls at the lowest dose of 0.1 mg TP/kg-bw/day, except in laboratory 4 when analyzed by either statistical approach and in laboratory 2 when using the Dunnett’s but not the *t*-test approach. There was a significant laboratory-to-laboratory effect in the responses (*R*^2^ = 36%). Laboratories 3 and 4 castrated the animals before PND 40 (PND 38 and PND 31, respectively) before preputial separation had occurred. In the pooled analysis, all TP doses led to significant increases in GP weight.

### COWS results

There were dose-dependent increases in the COWS weights in all laboratories. At the lowest dose of 0.1 mg/kg-bw/day, the weights were statistically significant except in laboratory 4. In laboratory 2, the weight increase was marginally statistically significant by the *t*-test and Dunnett’s tests when the starting body weights were used as the covariate, but not when the terminal body weights were used. The COWS weights had higher CVs than the other tissues. Because CVs were greatest in the vehicle group where the absolute weights were the smallest, the excision and weighing of the small COWS tissues may be technically demanding. In the pooled analysis, all TP doses led to a significant increase in COWS weights.

### Results of other studies and analyses

Two laboratories tested whether the corn oil itself could induce weight changes in the accessory sex tissues by including an untreated control group for comparison with the vehicle control group that received 0.5 mL corn oil/kg-bw. There were no effects on androgen-responsive tissues, other organ weights, or total body weights as a result of corn oil administration (data not shown).

There were dose-related weight changes in tissues other than those in the sex accessory organs. There were small, dose-related absolute increases in body weights with the TP treatment. These increases were consistent across all laboratories, but none achieved statistical significance (data not shown). Overall, there were increases in absolute kidney and liver weights as a function of TP dose, and a dose-related reduction in absolute adrenal weights (data not shown). However, these weight changes in these tissues did not achieve statistical significance in any of the laboratories. Overall liver and kidney weight CVs were 7.8 and 7.3%, respectively, whereas the adrenal weight CV was 12.3%, suggesting that adrenal dissection was more technically challenging.

All but one of the laboratories compared the weights of fresh and subsequently fixed (24-hr) VP. Three laboratories performed additional experiments to examine fixation of the SVCG and COWS, and one laboratory weighed the fixed adrenal glands. Fixation did not affect or improve the ability of any laboratory to detect dose-related increases in tissue weights at any of the TP doses (data not shown). After a discussion of these results, the VMG agreed to continue investigating the utility of fixation and included the fixed VP weight end point in the phase 1B protocol.

Two laboratories also and weighed the freshly excised the dorsolateral prostate, and three laboratories also weighed the fixed tissue. As with the other male sex accessory tissues, there was a significant dose-related increase in the weights of both the fresh and fixed dorsolateral prostate at all TP doses (data not shown). The VMG discontinued the work with the dorsolateral prostate and did not include it in the phase 1B protocol.

Four laboratories measured serum T and luteinizing hormone (LH) levels. The measured T concentrations were highly variable within and among laboratories at all levels of TP dosing. The analytical method was insufficient to detect T increases at the lowest test dose of 0.1 mg TP/kg-bw/day, where all sex accessory tissues were significantly increased in weight. Measured LH levels were also highly variable and less sensitive than the tissue weights to TP levels (data not shown). Therefore, the VMG discontinued the work with serum T and LH levels in phase 1B.

BMDs were calculated for each tissue within each laboratory and across laboratories. The across-laboratories BMDs are reported for each tissue along with the average CV (Table 2). In phase 1A, the BMDs for all tissues were within a 2- to 3-fold range, although the BMDs for the VP, SVCG, and COWS were somewhat lower than for the LABC and GP, whereas the CVs for the VP, SVCG, and COWS were higher than the LABC and GP (Table 2).

## Results of Phase 1B: Co-Administration of Selected FLU Doses with Reference TP Doses

In phase 1B, seven laboratories examined the ability of FLU to block the androgenic responses to TP. Four of the laboratories used doses of 0.2 and 0.4 mg, two laboratories used only 0.2 mg, and one laboratory used only 0.4 mg TP/kg-bw/day. All laboratories provided summaries of the protocols used and detailed Excel spread sheets containing the protocol information and test data. The laboratory means and standard deviations for each tissue for phase 1B are provided in Supplemental Material (http://www.ehponline.org/docs/2006/8751/suppl.pdf).

### VP results

**Figure 2:**
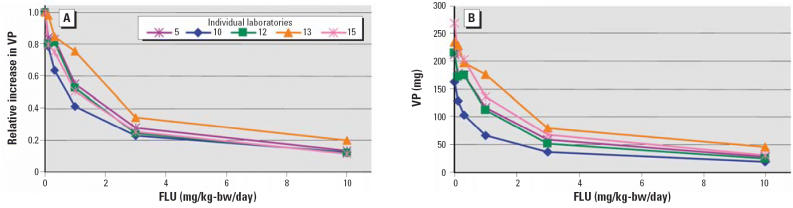
Response of VP weight to a reference dose of 0.4 mg TP/kg-bw/day with co-administration of FLU doses (mg/kg-bw/day) in phase 1B. (*A*) Relative (fold) decreases in the VP in all participating laboratories in surgically castrated male rats with a TP reference dose (subcutaneous) and co-administration of FLU doses (oral gavage) for 10 consecutive days and necropsy on day 11. (*B*) Absolute decreases in the VP in all participating laboratories in surgically castrated male rats with TP (subcutaneous) and co-administration of FLU doses (oral gavage) for 10 consecutive days and necropsy on day 11.

FLU significantly inhibited the effects of 0.2 and 0.4 mg TP/kg-bw/day on the VP in all laboratories in a dose-related manner. In animals treated with 0.2 TP mg/kg-bw/day, FLU significantly reduced the TP-stimulated VP weight gain at either 0.3 or 1 mg FLU/kg-bw/day depending on the statistical approach used. With 0.4 mg TP/kg-bw/day, using the *t*-test statistical approach, FLU induced a significant reduction in the TP-stimulated VP weight gain at doses between 0.1 and 1.0 mg/kg with the *t*-test statistical approach, and between 0.3 and 3 mg FLU/kg-bw/day with Dunnett’s test. At 10 mg FLU/kg-bw/day, the absolute VP weights approached the vehicle control (no TP) weights. In the pooled analyses, VP weight was significantly decreased at the 0.2-mg TP/kg-bw/day dose at 0.3 mg FLU/kg-bw/day and above. At 0.4 mg TP/kg-bw/day, the VP weight was significantly decreased at 0.1 mg FLU/kg-bw/day and above. The VP results for phase 1B are presented graphically in Figure 2, both as absolute weights and relative to the vehicle control.

### SVCG results

FLU significantly inhibited the effects of 0.2 and 0.4 mg TP/kg-bw/day on the SVCG in all laboratories in a dose-related manner. With both 0.2 and 0.4 mg TP/kg-bw/day, FLU induced a significant reduction in the stimulated VP weight gain between doses of 0.1 and 1 mg FLU/kg-bw/day, regardless of the statistical approach used. At 10 mg FLU/kg-bw/day, the absolute SVCG weights approached the vehicle control (no TP) weights. In the pooled analysis, the SVCG weight was significantly decreased at 0.3 mg FLU/kg-bw/day and above in both the 0.2 and 0.4 mg TP/kg-bw/day groups.

### LABC results

FLU significantly inhibited the effects of 0.2 and 0.4 mg TP/kg-bw/day on the LABC in all laboratories in a dose-related manner. With 0.2 mg TP/kg-bw/day, FLU induced a significant reduction in the TP-stimulated LABC weight gain in laboratory 5 at a dose of 0.3 mg FLU/kg-bw/day when the *t*-test was used. Otherwise, significance was reached only 1 mg/kg-bw/day; however in laboratory 13, the Dunnett’s test indicated marginal significance when starting weights were used, but not terminal weights, which had decreased. With 0.4 mg TP/kg-bw/day, FLU induced a significant reduction in the stimulated LABC weight gain between doses of 0.3 and 1 mg FLU/kg-bw/day regardless of the statistical approach used. At 10 mg FLU/kg-bw/day, the absolute LABC weights approached the vehicle control (no TP) weights. In the pooled analysis, LABC weight was significantly decreased at 0.3 mg FLU/kg-bw/day and above in the 0.2 mg TP/kg-bw/day groups and at the lower 0.1 mg FLU/kg-bw/day dose and above with the 0.4 mg TP/kg-bw/day groups.

### GP results

FLU significantly inhibited the effects of 0.2 and 0.4 mg TP/kg-bw/day on the GP in all laboratories in a dose-related manner. With 0.2 mg TP/kg-bw/day, FLU induced a significant reduction the TP-stimulated GP weight gain over a rather wide range from 0.1 and 3 mg FLU/kg-bw/day. The weights in laboratories 15 and 17 reached statistical significance at the lower FLU doses, where the GP weights in the TP-only group had higher starting weights. With 0.4 mg TP/kg-bw/day, FLU induced a significant reduction in the stimulated GP weight gain in the dose range of 1–3 mg FLU/kg-bw/day. At 10 mg FLU/kg-bw/day, the absolute GP weights approached the vehicle control (no TP) weights. In the pooled analysis, GP weight gain was significantly decreased at 0.3 mg FLU/kg-bw/day and above in the 0.2 mg TP/kg-bw/day groups, and beginning with the 0.1 mg FLU/kg-bw/day dose in the 0.4 mg TP/kg-bw/day TP groups.

### COWS results

FLU significantly inhibited the effects of 0.2 and 0.4 mg TP/kg-bw/day on the COWS in all laboratories in a dose-related manner. With 0.2 TP mg/kg-bw/day, FLU induced a significant reduction the TP-stimulated COWS weight gain between of 0.3 and 3 mg/kg-bw/day. With 0.4 mg TP kg-bw/day, FLU induced a significant reduction in the stimulated COWS weight gain in the dose range of 0.1–3 mg FLU/kg-bw/day. At 10 mg FLU/kg-bw/day, the absolute COWS weights approached the vehicle control (no TP) weights. In the pooled analysis, COWS weight was significantly decreased at 0.1 mg FLU/kg-bw/day and above in the 0.2 mg TP/kg-bw/day groups and at the 0.3 mg FLU/kg-bw/day and above doses in the 0.4 mg TP/kg-bw/day groups.

### Results of other measurements

As in phase 1A, treatment of the rats with 0.2 or 0.4 mg TP/kg-bw/day for 10 days resulted in low, but consistent, absolute body weight gains that were not statistically significant at either dose (data not shown). The administration of FLU to the TP-treated rats led to lower body weights than in the TP-only treated animals after the 10-day treatment period, but the differences were not statistically significant (data not shown). FLU treatment did not affect the modest absolute increases in liver and kidney weight gains induced by TP, but FLU treatment mitigated the TP-induced decrease in adrenal weight gains in a dose-related manner (data not shown). All increases in recorded adrenal weight were significant at the 10 mg/kg-bw/day FLU dose.

Several laboratories in phase 1B continued to compare the impact of fixation on the VP weights. Statistical analyses of the fixed VP weights resulted in the same dose–response sensitivity as the fresh VP weights, and fixation of the VP did not consistently affect the CV of the measurement within or among laboratories.

The sensitivities of each of the tissues to FLU inhibition of TP-induced weight gain in phase 1B were evaluated using the BMD approach. The BMDs for all tissues were within a 2- to 3-fold range, and the BMDs did not consistently favor either the 0.2 or 0.4 mg TP/kg-bw/day reference doses (Table 3).

## Discussion and Conclusions

The regulatory need for the Hershberger bio-assay is to identify and to assist in the prioriti-zation of test substances that may have an androgenic or antiandrogenic mechanism of action. The androgen receptor is the molecular starting point for both adverse effects in the male reproductive tract during *in utero* development and the control of growth responses of the male sex accessory tissues (as measured in the Hershberger bioassay). Further, a substance’s androgen receptor affinity and complex absorption, transformation, distribution, and excretion processes should be sufficiently similar between *in utero* and peripubertal exposures to support the relevance of the assay as an *in vivo* screen for regulatory concerns. The short time frame of the test and the limited number of animals needed lend further support to the assay. These rationales for the regulatory use of the Hershberger assay have been broadly supported in scientific workshops (European Commission/OECD 1997; Gray et al. 1997).

The primary purpose of this phase 1 validation effort was to test the robustness and reproducibility of the Hershberger protocol developed for the OECD. In that regard, the data presented here support the conclusion that the Hershberger assay is measuring relevant biological responses; that it is sufficiently sensitive, robust, and reproducible to detect androgenic and antiandrogenic activities; and that the protocol is adequate to proceed to the next phase of validation. All laboratories were successful in detecting changes in the weights of TP-stimulated male sex accessory glands with the standardized protocol, and the inhibition of weight gain in TP-stimulated tissues when the antagonist FLU was co-administered. In phase 1A, all five of the androgen-sensitive sex accessory tissues increased in weight as a function of TP dose in all laboratories. In phase 1B, the antagonistic activity of FLU to TP was detectable in all five of the androgen-sensitive sex accessory tissues in a dose-related manner with both reference doses of TP. The use of six animals per dose group was sufficient to robustly detect the androgenic and anti-androgenic activity of potent substances.

The next phase of the validation program will determine if six animals per group will be sufficient for detecting weaker androgens and antiandrogens. The ability of the protocol to detect these changes was not affected by differences in rat strain, diet, caging, routine laboratory procedures, and modest differences in the ages at which the animals were castrated. The data did suggest that the animals should be castrated after preputial separation occurs (usually after 42 days). When the animals were castrated earlier, the GP was not fully separated from the prepuce in some laboratories, making it more difficult to dissect and increasing the CV in these cases.

The use of slightly different statistical approaches to analyze the data yielded consistent results. As was expected, the *t*-test approach was occasionally able to reach statistical significance when the Dunnett’s approach remained marginally insignificant. The test results were also evaluated further by an independent statistician, and the statistical outcomes and results were reproducible. The independent analysis tested the ANOVA assumptions, that is, that the error terms are independently normal with constant variance. Because only a log transformation was used originally, the independent analysis also investigated the use of untransformed data or a square-root transformation procedure. Among the various transformations examined, the log transformation was the best overall method. However, data transformation was not necessary in all cases, and no single transformation was consistently better in normalizing the data across all laboratories and end points [Supplemental Material (http://www.ehponline.org/docs/2006/8751/suppl.pdf)]. In a few isolated cases, the lowest observed effect level (LOEL) value changed when the more appropriate transformation procedure (i.e., absence of transformation in all cases) rather than log transformation was used [Supplemental Material (http://www.ehponline.org/docs/2006/8751/suppl.pdf)]. Such changes were few and minor, the LOELs remained consistent across laboratories and end points, and the additional independent statistical analysis supports the claim that the Hershberger assay is robust.

The utility and sensitivity of the five mandatory sex accessory tissues were evaluated. Statistical significance is used to yield a LOEL dose, and statistical significance is largely determined by a combination of the degree or percentage of change, and the degree of variability, in the measurement. First, these tissues undergo very different overall relative changes in weight from > 10-fold (1000%) for the VP and the SVCG) to 80–120% for the GP [Supplemental Material (http://www.ehponline.org/docs/2006/8751/suppl.pdf)]. Second, the tissues differ in size, whether they contain fluid, and other aspects related to potential difficulties and variability during tissue dissection, handling, and weighing. These differences should be collectively described by the CV value for the different tissues, and these values vary considerably. The CVs for starting and terminal body weights and the five mandatory sex accessory tissues have been summarized for phase 1A and phase 1B, leading to three conclusions: *a*) the CVs for fluid-filled tissues (VP, SVCG, COWS) are approximately double those for the solid tissues (LABC and GP), and *b*) some laboratories consistently have lower CVs, suggesting proficiency differences in dissection. Similar associations of CV and laboratory proficiency have been noted during the uterotrophic validation (Kanno et al. 2001, 2003). In the case of the male sex accessory tissues themselves, the tissues with the highest responses also have the highest CVs, and vice versa, which suggests that no large differences in sensitivity may exist among the tissues.

An evaluation of the LOELs for the different tissues supports this hypothesis. No dramatic differences were noted in the LOELs of the different tissues. In phase 1A, except for laboratories with relatively high CVs, all tissues achieved statistical significance at the lowest TP dose of 0.1 mg/kg-bw/day. In phase 1B, the tissues achieved statistical significance in a similar range of FLU doses, and most LOELs were in 0.3–1 mg FLU/kg-bw/day range with either 0.2 or 0.4 mg TP/kg-bw/day as the reference dose [Supplemental Material (http://www.ehponline.org/docs/2006/8751/suppl.pdf)]. When the tissues are compared by the number of times they provided or were coincident in providing the LOEL dose, the VP did so in three instances, the SVCG in six instances, the LABC once, the GP once, and the COWS in four instances.

BMDs were calculated for each tissue along with its average CV for phase 1A (Table 2) and phase 1B (Table 3). The BMDs were somewhat lower for the VP, SVCG, and COWS than the LABC and GP in phase 1A but were not distinguishable overall in phase 1B. Therefore, without compelling technical support that one tissue is distinctly less sensitive than the others, all five tissues will continue to be required in the phase 2 protocol using additional agonist and antagonist test substances.

In terms of the other measurements and procedures, no added value could be attributed to measurements of the dorsolateral prostate, the fixation of any of the tissues, or serum hormone (T or LH) analyses. Therefore, these measures will not be included in phase 2 of the validation program. Liver, kidney, and adrenal weights will be retained as optional measurements in phase 2.

To summarize, all laboratories were successful in detecting increases in androgen-responsive tissue weights using TP, and decreases in TP stimulated tissue weight when FLU was co-administered. The basic protocols performed well under a variety of different laboratory conditions (e.g., strain, diet, housing protocol, bedding, vehicle). There was good agreement among laboratories with regard to the lowest effect TP doses inducing significant increases in tissue weights and to the FLU doses inducing decreases in TP-stimulated tissue weights. Several additional procedures (e.g., fixation of certain tissues before weighing) and serum component measurements (e.g., serum hormones) were also included in these studies to assess their potential utility but have not been included in the final protocols.

In conclusion, the results indicated that the OECD Hershberger protocols were robust, reproducible, and transferable across laboratories when using high-potency compounds such as TP and FLU. The protocols have been refined, and the next phases of the OECD validation program will test the protocol with selected doses of weak androgen agonists, androgen antagonists, a 5α-reductase inhibitor, and chemicals having no androgenic activity.

## Figures and Tables

**Table 1:** Measurements recorded by individual participating laboratories in phase 1 of the OECD Hershberger validation program.

	Laboratory															
Dose response	1	2	3	4	5	6	7	8	9	10	12	13	14	15	16	17
TP dose response																
Mandatory tissues^a^	Y^b^	Y	Y	Y	Y	Y	Y	Y	Y	Y	Y	Y	Y	Y	Y	Y
Kidney			Y	Y	Y	Y	Y	Y		Y	Y	Y	Y	Y		
Adrenals	Y		Y	Y	Y	Y	Y			Y	Y	Y	Y			
Dorsolateral prostate														Y		Y
VP (fixed)										Y						Y
Dorsolateral prostate (fixed)										Y				Y		Y
SVCG (fixed)										Y						Y
COWS (fixed)										Y	Y					Y
Serum hormones^c^			Y								Y					Y
Untreated group^d^				Y	Y											
FLU dose response																
Mandatory tissues^a^						Y		Y		Y^b^	Y	Y^b^		Y	Y	Y^b^
Kidney						Y		Y			Y			Y	Y	Y
Adrenals						Y					Y			Y	Y	Y
Dorsolateral prostate										Y						Y
Fixed tissues											Y^e^					Y^f^
Serum hormones^b^											Y					

Y, yes.

a VP (fresh tissue, and fixed), dorsolateral prostate, SVCG LABC, GP, COWS, daily body weights, and liver weights.

b No fixed VP weight recorded.

c T and LH analyses in serum.

d These laboratories included an additional untreated control group (no vehicle was administered to this group).

e COWSs were fixed.

f VP, SVCG, dorsolateral prostate, and COWS were fixed.

**Table 2:** BMDs (mg TP/kg-bw/day) for male sex accessory tissues in phase 1A.

	BMD (95% lower confidence limit on the BMD)		
Tissue	Log_10_ transformation	“Correct”transformation^a^	Average CV (%)
VP	0.066 (0.061)	0.089 (0.080)^b^	23.67
SVCG	0.083 (0.077)	0.120 (0.107)^c^	20.04
LABC	0.184 (0.152)	0.310 (0.256)^b^	12.92
GP	0.226 (0.196)	0.256 (0.189)^b^	12.79
COWS	0.079 (0.0676)	0.129 (0.112)^c^	22.62

a If the untransformed or a square-root transformation gave a larger (nonsignificant) *p*-value for normality using Wilk-Shapiro, then the alternative transformation was applied and a BMD on that data set was calculated.

b Untransformed data.

c Square-root transformation.

**Table 3:** BMDs (mg FLU/kg-bw/day) for male sex accessory tissues in phase 1B.

		BMD (95% lower confidence limit on the BMD)		
Tissue	TP dose (mg/kg-bw/day)	Log_10_ transformation	“Correct” transformation^a^	Average CV (%)
VP	0.2	0.603 (0.512)	0.499 (0.418)^b^	20.87
	0.4	0.609 (0.525)		
SVCG	0.2	0.542 (0.477)		20.93
	0.4	0.510 (NA)	0.311 (0.271)^b^	
LABC	0.2	1.115 (1.007)	0.917 (0.790)^b^	11.35
	0.4	0.501 (NA)	0.293 (0.240)^c^	
GP	0.2	0.502 (NA)	0.332 (0.218)^c^	9.36
	0.4	1.308 (NA)	1.067 (0.825)^b^	
COWS	0.2	1.333 (NA)		19.69
	0.4	0.948 (0.737)		

NA, lower bound computation did not converge.

a If the untransformed or a square-root transformation gave a larger (nonsignificant) *p*-value for normality using Wilk-Shapiro, then the alternative transformation was applied and a BMD on that data set was calculated.

b Square-root transformation.

c Untransformed data.
