# Supplementary material for: The OECD Program to Validate the Rat Hershberger Bioassay to Screen Compounds for in Vivo Androgen and Antiandrogen Responses. Phase 1: Use of a Potent Agonist and a Potent Antagonist to Test the Standardized Protocol
Source: Environ Health Perspect. 2006 Feb 27;114(8):1259–65. doi: 10.1289/ehp.8751 (PMC1552005; doi:10.1289/ehp.8751)

EHP 8751: OECD Validation of Rat Hershberger Assay: Phase-1

SUPPLEMENTARY MATERIALS

These Supplementary Materials are arranged in 4 sections:

I. A descriptive outline of the Hershberger protocol.
II. The dissection guidance provided to the laboratories.
III. A set of detailed data tables including means, standard deviations, and coefficients of variation for each laboratory as well as group calculations for each chemical dose group.
IV. An example of the statistical output for one of the Dunnett's analyses.

Section I. A descriptive outline of the Hershberger protocol.

The original protocol was drafted by the Lead Laboratory (L. Earl Gray, Jr, USEPA). This was submitted to the OECD Secretariat and distributed to the VMG-mammalian for comments. The revised protocol was then distributed to both independent experts and some 15 laboratories that had agreed to participate in-Phase 1A. The finalized protocol outline, intended to allow some flexibility for individual laboratory preferences, can be found in the

1) the use of peripubertal male rats because the rat is commonly used for the higher tier reproductive and developmental assays (only the Fisher 344 strain was excluded; because of its high spontaneous testicular tumor rates);
2) castration in the time period of postnatal days (pnd) 35-42 days;
3) a post-castration recovery period of 7-14 days;
4) initiation of test substance administration before pnd 50;
5) the use of general animal husbandry conditions: room temperature of 22 ± 3 C^{o}; a relative humidity 30-70%; artificial lighting with a 12 hour light and 12 hour dark cycle; and feed and drinking water (tap or filtered) provided *ad libitum*;
6) daily administration of test substances for ten consecutive days with necropsy approximately 24 hours after the last administration;
7) group sizes of 6 animals and the inclusion vehicle control group;
8) subcutaneous administration of TP on the shaved dorsal surface;
9) oral gavage administration of FLU;
10) the use of corn oil as the vehicle;
11) limiting the maximum dosage volumes to 0.5 mL/kg-bw/d for TP and 5 mL/kg-bw/d for FLU;
12) random assignment of the animals among the groups to achieve approximately equivalent mean body weights;
13) mandatory daily measurements of all clinical signs, individual body weights to 0.1 g, and the volume(s) of test substance solutions administered;
14) mandatory measurements at necropsy of tissue and organ weights, to 0.1 mg, of the VP (both fresh and after 24 hrs fixation), the paired SVCG, the GP, the LABC, and the paired Cowper's (also known as bulbourethral) glands (COWS), and, to 0.1 g, of the liver and total body weights; and
15) inclusion of a number of optional measurements and procedural options for investigation and comparison including the dorso-lateral prostate weight (DL-P), the paired adrenal gland weights, the paired kidney weights, the fixation weights of tissues other than the VP, a naïve control without the administration of vehicle (to assess any possible changes from substances in the corn oil vehicle or due to its caloric content), and serum levels of testosterone (T) and luteinizing hormone (LH) at the time of sacrifice.

Section II. The dissection guidance provided to the laboratories.

Dissection Guide for the Hershberger Assay With the Castrate Immature Male Rat

Prepared by the Lead Laboratory for the Hershberger Validation Program, MARCH, 2000
L. Earl Gray, Jr., Johnathan Furr and Jsoeph Ostby
Address:
Endocrinology Branch, RTD, NHEERL, ORD, MD-72
United States Environmental Protection Agency
Research Triangle Park, NC, 27711, USA
Phone: 919-541-7750 Fax: 919-541-4017 E-Mail: gray.earl@epa.gov

DISCLAIMER. The information contained in this document does not necessarily reflect NHEERL or USEPA policy.

OUTLINE OF DISSECTION GUIDE

1. BACKGROUND FIGURES OF THE REPRODUCTIVE ORGANS OF THE ADULT MALE RAT. (**Figures 1-5**) – Emphasis on the muscle identification and dissection.
2. SEQUENTIAL NECROPSY DESCRIPTION WITH REFERENCE TO PHOTOS
   a. Anesthetize the animal (if serum is to be collected).
   b. Collect serum by cardiac puncture (optional).
   c. Euthanize humanely, decapitate, exsanguinate and place animal on dissection board with the ventral surface upwards (**see Photo 1**).
   d. Determine if the prepuce of the penis has separated from the glans penis. If so, then retract the prepuce and remove the glans penis and weigh (nearest 0.1 mg) (**see Photo 2**). The glans penis in this photo weighed 67.5 mg.
   e. Remove abdominal skin and muscle layers, exposing viscera.
   f. Remove liver, stomach, intestines, kidney, adrenals, etc. Weigh liver to nearest 0.1 g, and the paired kidneys and paired adrenals to the nearest 0.1 mg. This dissection then exposes the seminal vesicles plus coagulating glands (SV) (**Photo 3, panel 1**), SV and bladder (B).
   g. Dissection of the Ventral Prostate. **Photo 3.**
      Separate bladder (B) from ventral muscle layer by cutting connective tissue (CT) along the midline with iris scissors (**Photo 3, panel 2**).

      Displace the bladder anteriorly towards the seminal vesicles (SV), revealing the left and right lobes of the ventral prostate (indicated by asterisks) covered by a layer of fat. Using fine tweezers (with blunt not sharpened tips) carefully tease the fat (F) layer from the right (**Photo 3, panel 3**) and left (**Photo 3, panel 4**) lobes of the ventral prostate.

      With a pair of tweezers in each hand, gently displace right lobe of the ventral prostate (asterisk) from the urethra (**Photo 3, panel 5**) and dissect this lobe of the ventral prostate from the urethra with scissors (**Photo 3, panel 6**).

      Still holding the right lobe of the ventral prostate with forceps, gently displace left lobe of ventral prostate away from the urethra (**Photo 3, panel 7**) and then dissect this lobe from the urethra with iris scissors and weigh to nearest 0.1 mg.

For comparison, Figure 2 displays a diagram of the sex accessory tissues of the adult male rat. Note that these are considerably larger than those in the immature male rats in Hershberger assays, including the protocol described herein. The ventral prostate in these photos were taken from a castrated (at 41 days of age)-immature (necropsied at 56 days of age)-TP-treated male SD rat and weighed 38.5 mg. In contrast, the ventral prostate of a six month old control SD male rat generally weighs more than 500 mg.

h. Dissection of the Seminal Vesicle (plus coagulating glands with fluid). (**Photo 4**).

Displace the bladder (B) caudally, exposing the vas deferens and right and left lobes of the seminal vesicles (SV) plus coagulating glands (**Photo 4, panel 1**). In **Photo 4, panel 2**, a paper towel has been placed under the SVs to enhance discrimination of these glands from the muscle and fat layers below. In **Photo 4, panel 3**, a hemostat is clamped at the base of the SVs, where the vas deferens join the urethra. This prevents leakage when the SVs are dissected with iris scissors from the urethra, as shown in **Photo 4, panel 4**. Photo 4, panel 5 displays the SVs held with the clamped hemostat against a dark background, demonstrating that these tissues will not leak fluid, if properly dissected. These tissues are place in a tared weigh-boat and trimmed of fat and adnexa, the clamp removed and the SV weighed to the nearest 0.1 mg. The SV in this photo weighed 162.0 mg, whereas the SV from an adult weighs over 1500 mg.

i. Dissection of the levator ani plus bulbocavernosus (LA-BC) muscles. (**Photo 5**)

The provided Figures 3, 4 and 5 provide background diagrams of the location of the LABC muscles. At the caudal end, the LA muscles wrap around the colon, while the anterior LA and BC muscles are attached to the penile bulbs (Figure 5).

These muscles and the base of the penis with penile bulbs are exposed (as shown in **Photo 5, panel 1**) by removal of the skin and adnexa from the perianal region extending from the base of the penis to the anterior end of the anus. When fat is carefully removed from these tissues with tweezers and iris scissors, these muscles are readily identifiable (**Photo 5, panel 2**) as per Figures 3, 4 and 5. As shown in **Photo 5, panel 3**, the BC muscle is grasped with blunt tweezers, while the muscle is dissected from the penile bulb such that the white connective tissue and "reddish" corpus spongiosum are detached from the BC muscles one each side. At this time, the BC muscles are lifted gently upward (**Photo 5, panel 4**) away from the body and the colon is cut in two with iris scissors. After the colon has been cut, the LABC can be pulled further upward and fat and adnexa can be pulled off with tweezers or cut off with scissors. When removed from the carcass (**Photo 5, panel 5**), the LABC should resemble a "ring" which can easily be trimmed of fat and adnexa and weighed to the nearest 0.1 mg. The LABC in this photo weighed 251.6 mg, whereas the LABC of the adult male is about 1200 mg.

After the LABC has been removed the round Cowper's or bulbourethral glands (CG) are visible at the base of, and slightly dorsal to, the penile bulbs. These are removed by careful dissection with iris scissors. One must avoid nicking the thin capsule, such that there is no leakage of fluid from the paired glands. These are weighed paired to the nearest 0.1 mg, unless one of the glands leaks during necropsy, in which case this should be noted and the glands should he weighed individually, noting which gland was weighed without leakage. The Cowper's glands in this photo weighed 18.1 mg, as compared to about 200 mg in an adult male.

Figure 1

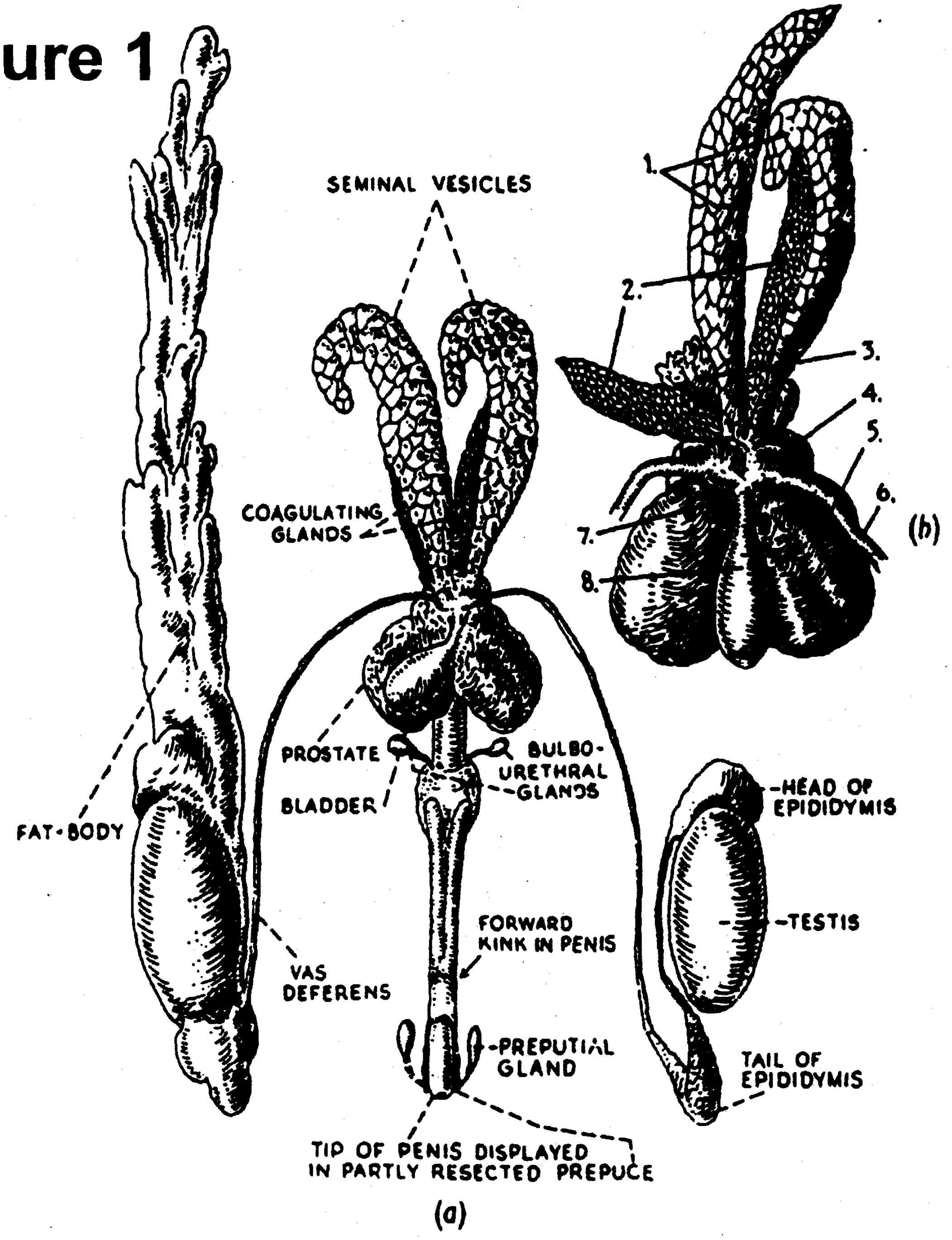

Reproductive organs of the male rat. (a): Anterior view with the epididymal fat body removed on the left side; (b): magnified view of the prostatic lobes and seminal vesicles with the right lobe of the coagulating gland drawn away from the seminal vesides to display the dorsolateral lobe of the prostate. 1, Seminal *vesicles; 2, coagulating* glands; 3, lobe of dorsolateral prostate; 4, ampullary gland; *5,* lobe *of* ventral prostate; 6, ductus deferens; 7, ureter; 8, bladder (displaced downwards). from: The Physiology of Reproduction Volume 1, second edition, Chapter 18, Setchell et al.

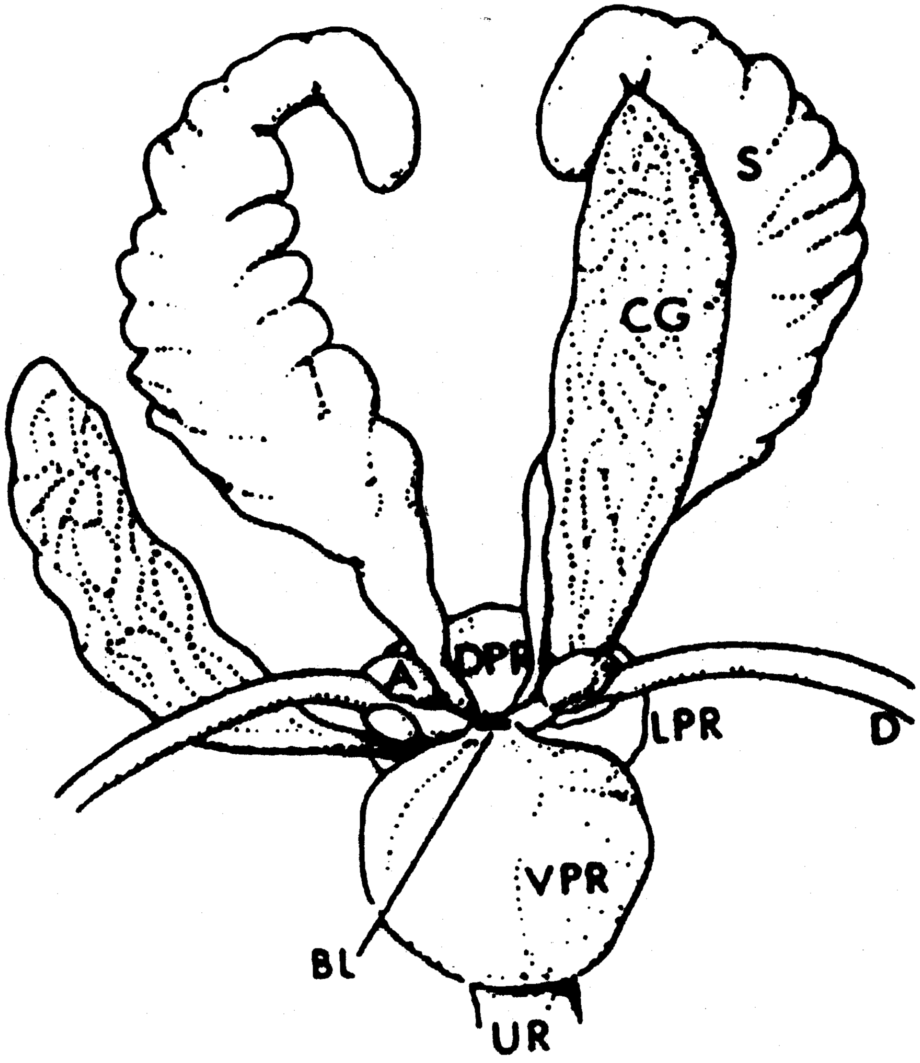

Figure 2 Male accessory reproductive glands - from: The Physiology of Reproduction Volume 1, second edition, Chapter 23, Luke and Coffey, 1991

**S.**	**seminal vesicle**
**CG.**	**coagulating gland**
**BL.**	**bladder**
**UR.**	**urethra**
**VPR.**	**ventral prostate**
**LPR.**	**lateral prostate**
**DPR.**	**dorsal prostate**
**A.**	**ampullary gland**
**D.**	**vas deferens.**

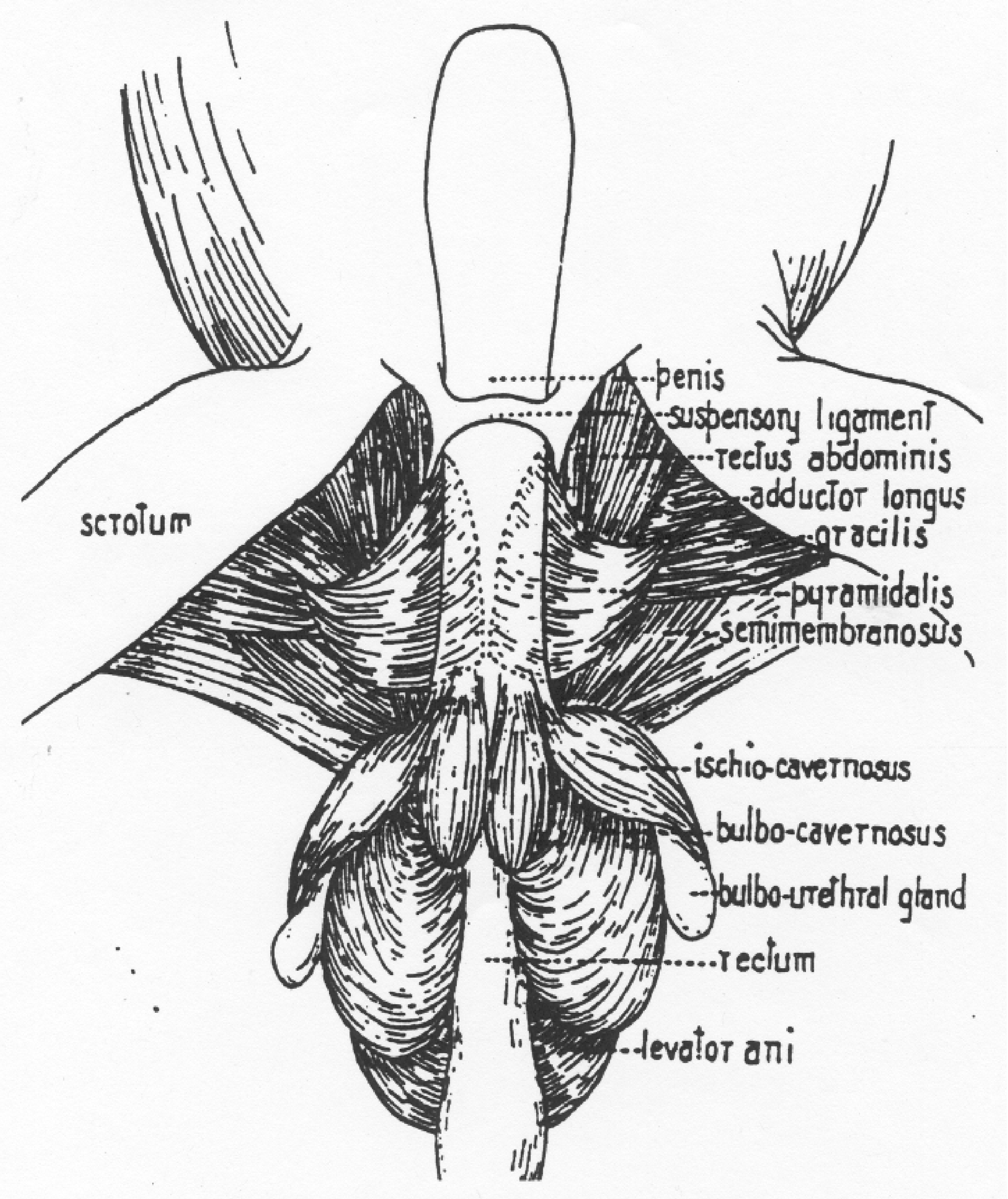

Figure 3 – Muscles of the male perineum - from Rand, M.N., and Breedlove, S.M. 1992. Androgen locally regulates rat bulbocavernosus and levator ani size. J Neurobiology. 23(1):17-30.

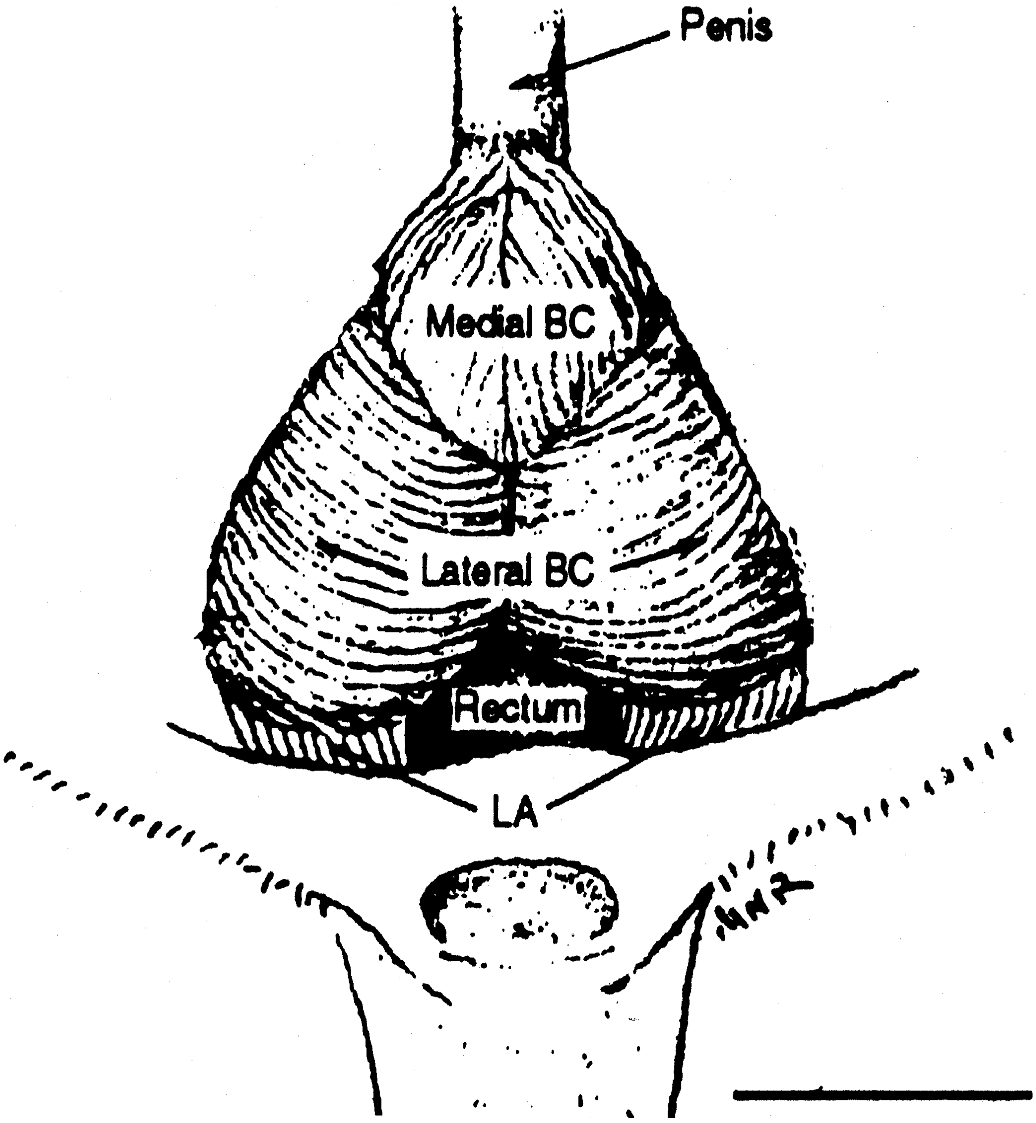

Figure 4. Muscles responsive to Androgens that will be dissected- from Rand, M.N., and Breedlove, S.M. 1992. Androgen locally regulates rat bulbocavernosus and levator ani size. J Neurobiology. **23(1):17-30.**

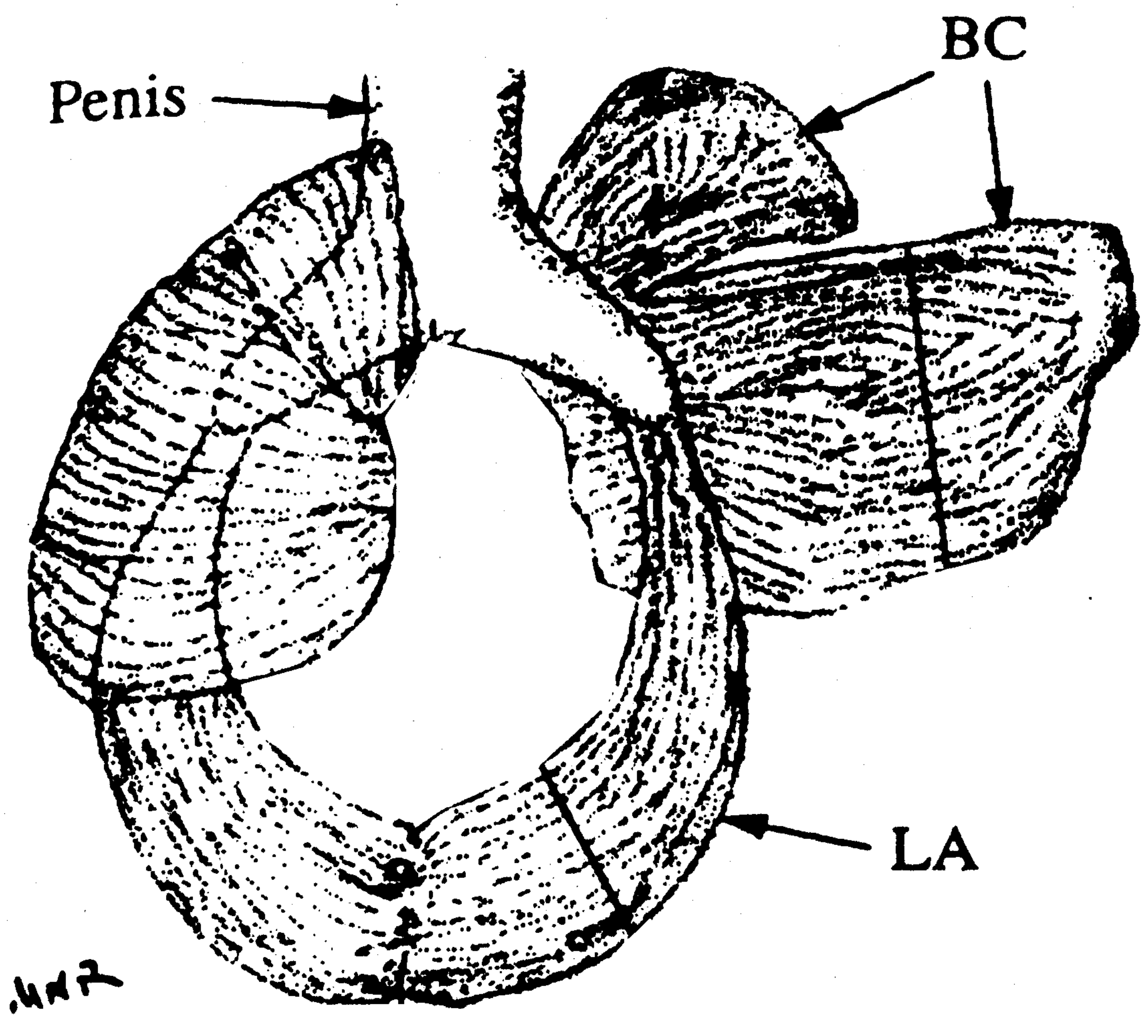

Figure 5. Illustration of the BC/ LA muscle complex after dissection and removal from the perineum. The animal's left BC is shown dissected away from the base of the penis and the LA. from Rand, M.N., and Breedlove, S.M. 1992. Androgen locally regulates rat bulbocavernosus and levator ani size. J Neurobiology. 23(1):17-30.

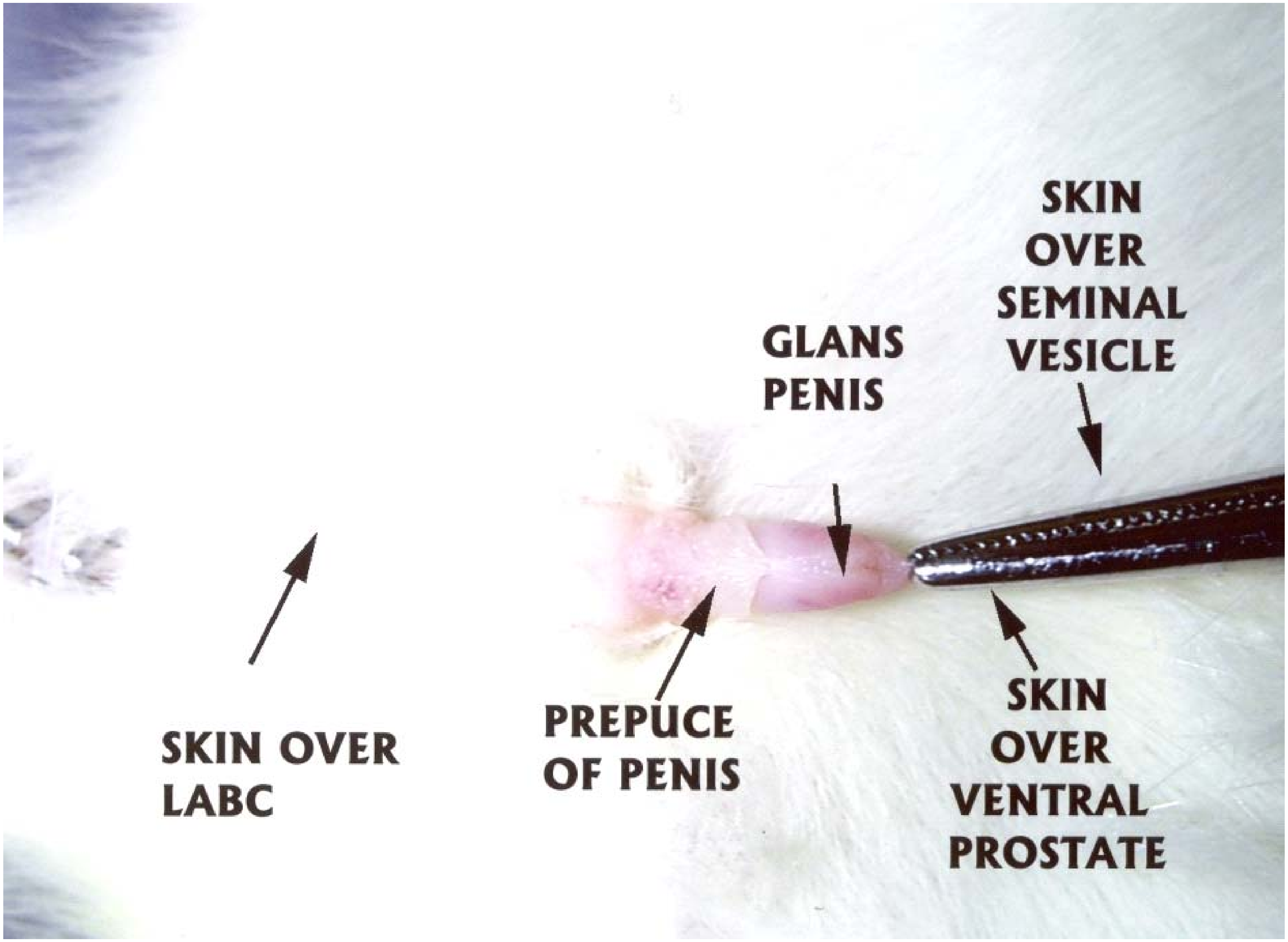
SKIN OVER SEMINAL VESICLE
GLANS PENIS
SKIN OVER LABC
PREPUCE OF PENIS
SKIN OVER VENTRAL PROSTATE

PHOTO 1

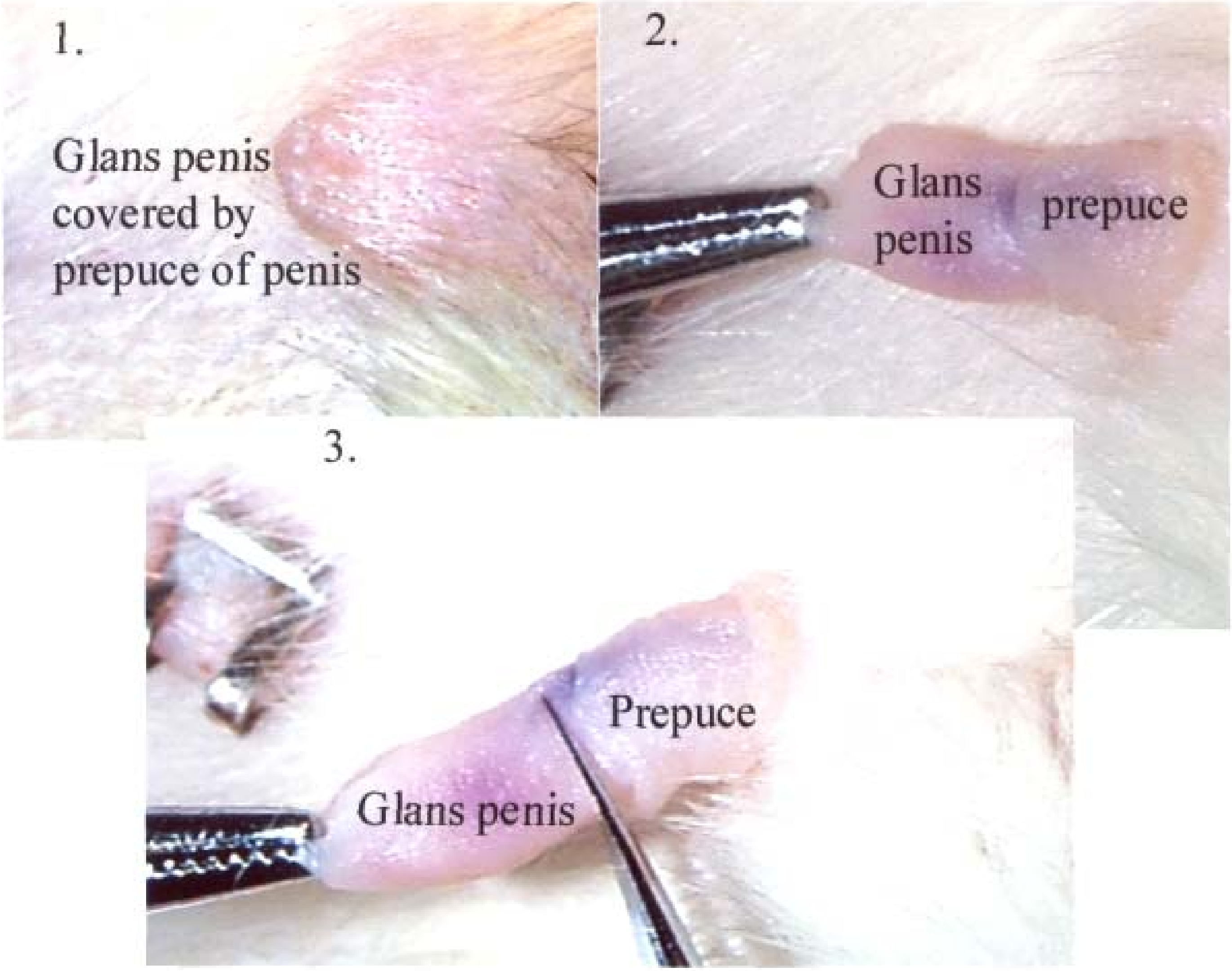
1.
Glans penis covered by prepuce of penis
2.
Glans penis
prepuce
3.
Prepuce
Glans penis

PHOTO 2

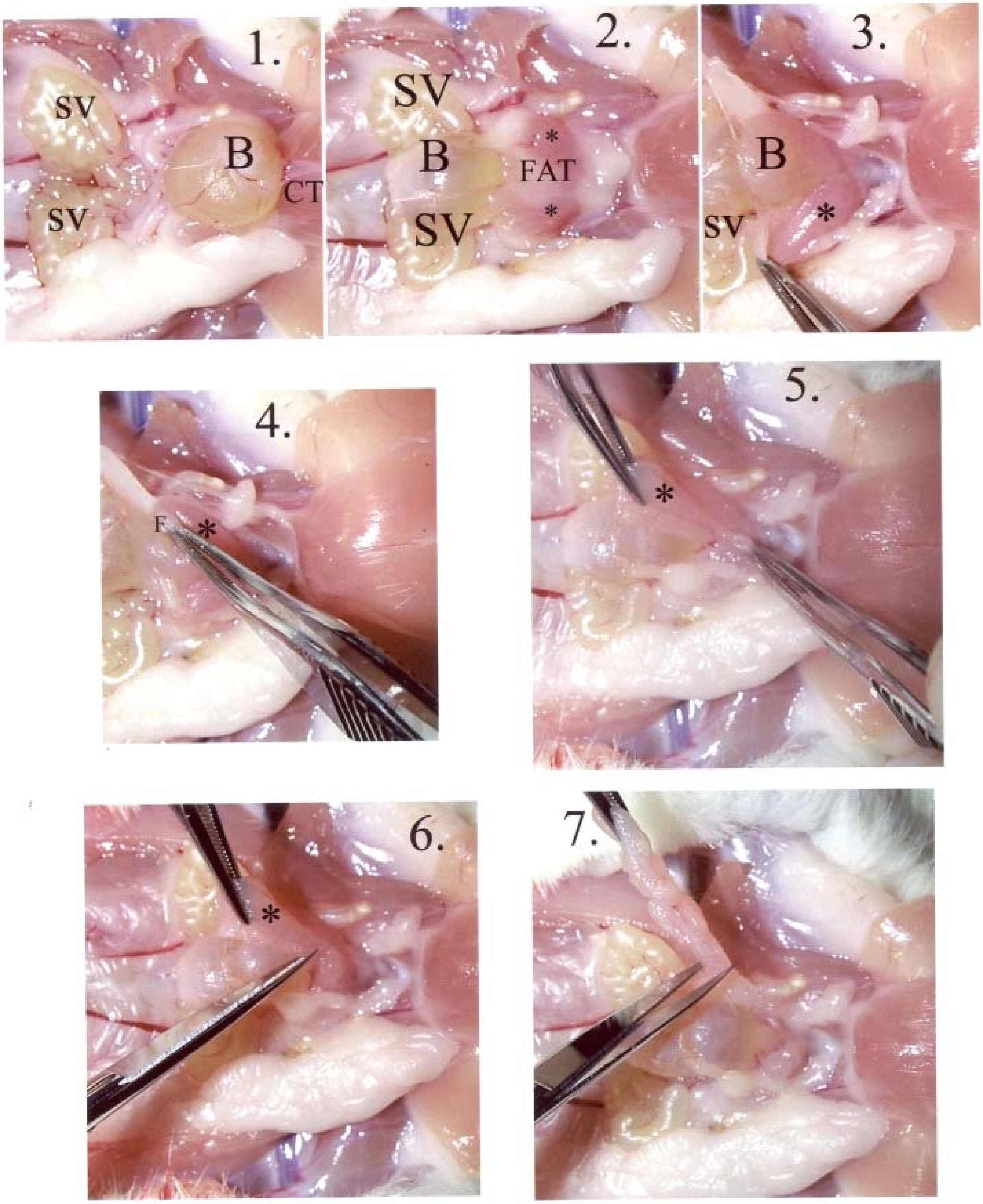

PHOTO 3

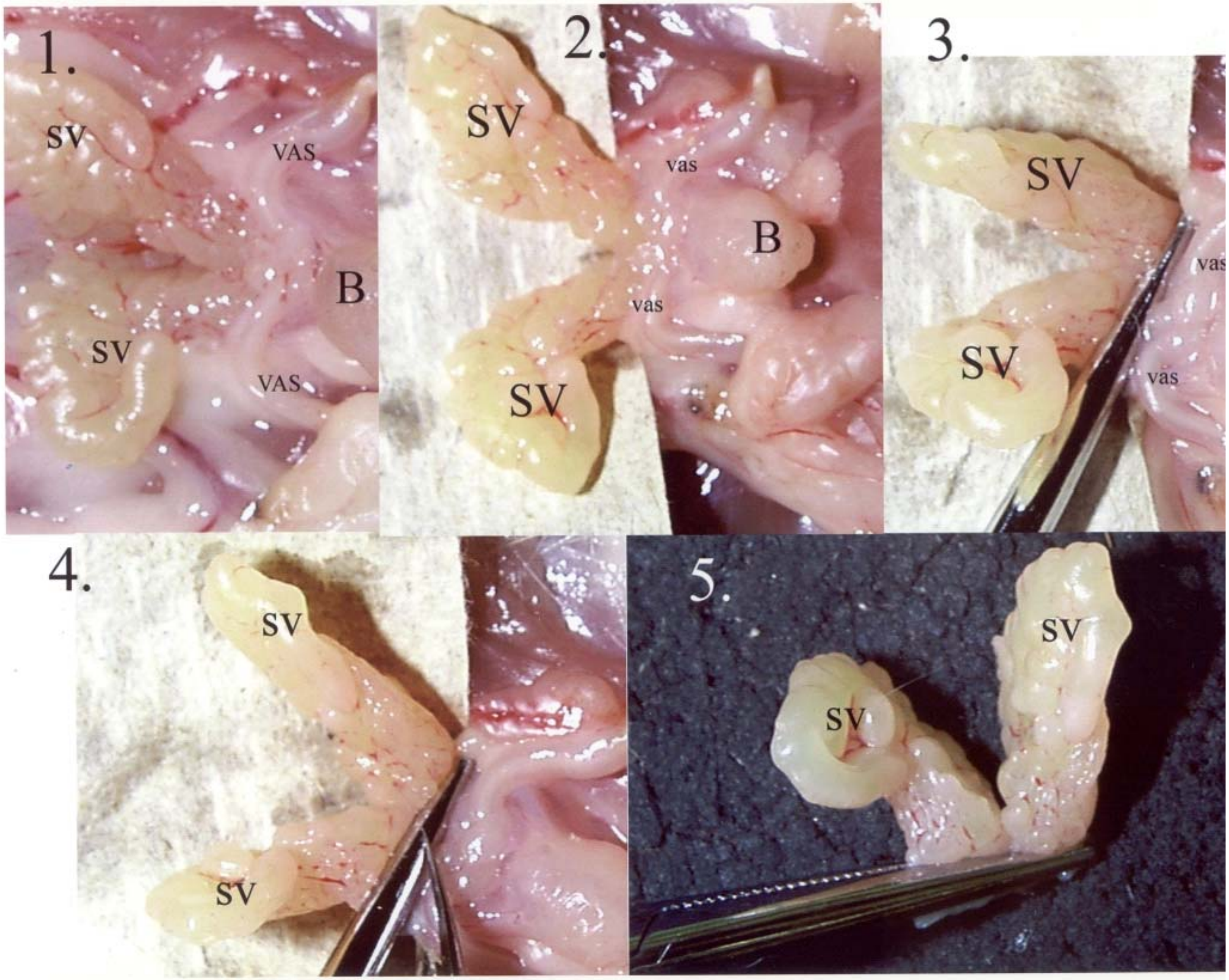

PHOTO 4

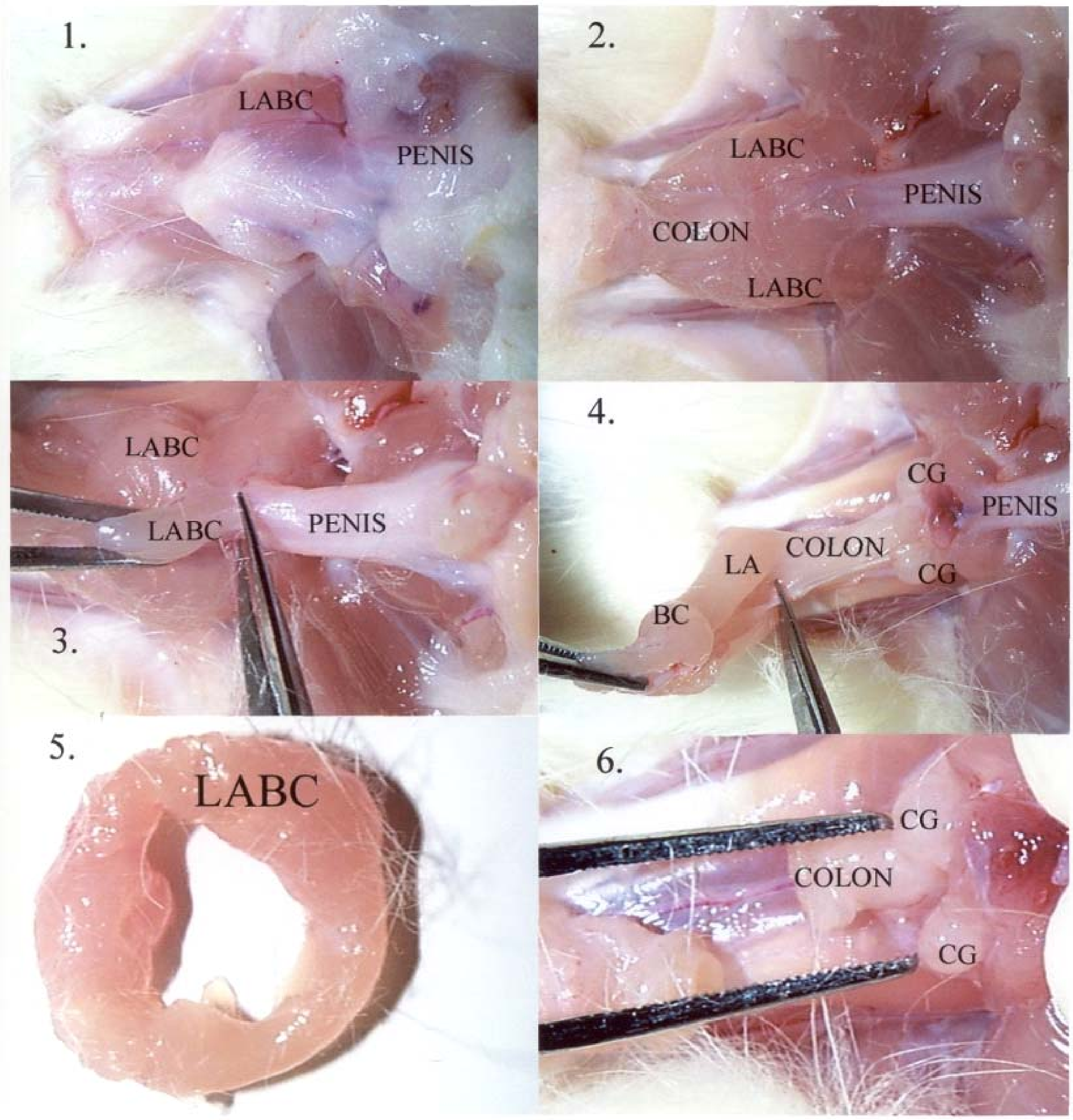
1.
LABC
PENIS
2.
LABC
PENIS
COLON
LABC
LABC
LABC
PENIS
3.
4.
CG
PENIS
COLON
LA
CG
BC
5.
LABC
6.
CG
COLON
CG

PHOTO 5

Section III. A set of detailed data tables including laboratory conditions as well as means, standard deviations, and coefficients of variation for each laboratory as well as group calculations for each chemical dose group.

This section contains the following tables of detailed data for Phase-1 of the OECD Hershberger Validation Program.

Suppl. Material Table 1. Rat strains and suppliers, animal ages, and husbandry conditions used for the Testosterone Propionate dose-response studies.

Suppl. Material Table 2. Rat strains and suppliers, animal ages, and husbandry conditions used for the Flutamide dose response studies.

Suppl. Material Table 3. Dose Response for Ventral Prostate in Phase-1A (mg, mean ± SD)

Suppl. Material Table 4. Dose Response for Seminal Vesicles and Coagulating Glands in Phase-1A (mg, mean ± SD)

Suppl. Material Table 5. Dose Response for Levator Ani and Bulbocavernosus in Phase-1A (mg, mean ± SD)

Suppl. Material Table 6. Dose Response for Glans Penis in Phase-1A (mg, mean ± SD)

Suppl. Material Table 7. Dose Response for Cowper's Glands in Phase-1A (mg, mean ± SD)

Suppl. Material Table 8. Dose Response for Ventral Prostate in Phase-1B (mg, mean ± SD)

Suppl. Material Table 9. Dose Response for Seminal Vesicles and Coagulating Glands in Phase-1B (mg, mean ± SD)

Suppl. Material Table 10. Dose Response for Levator Ani and Bulbocavernosus Muscles in Phase-1B (mg, mean ± SD)

Suppl. Material Table 11. Dose Response for Glans Penis in Phase-1B (mg, mean ± SD)

Suppl. Material Table 12. Dose Response for Cowper's Glands in Phase-1B (mg, mean ± SD)

Suppl. Material Table 13. Evaluation of statistical transformations used to normalise the Phase-1A data

Suppl. Material Table 14. LOEL changes in Phase-1A as an effect of data transformation used

Suppl. Material Table 15. Coefficients of Variation for Body Weights and Male Accessory Tissues in Phase-1A

Suppl. Material Table 16. Coefficients of Variation for Body Weights and Male Accessory Tissues in Phase-1B

Suppl. Material Table 1. Rat strains and suppliers, animal ages, and husbandry conditions used for the Testosterone Propionate dose-response studies.

LAB	Rat Strain and Supplier	Bedding material	Diet and Supplier	Age at Castration (Days)	% Controls With PPS	Starting Weight (gms)	Rats per cage
1	Alpk:APfSD; on-site animal breeding facility	Paper (shredded coffee filter)	R&M No. 1 Special Diet Services Ltd., batch 6458	42-44	100	224	3
2	Sprague-Dawley; Iffa Credo, France	Suspended steel wire mesh	Pietrement aliment type M20; Lot no. 991125	33-47	100	271	1
3	CRL:WI(GLX/BRL/HAN) IGS BR; Charles River, GR	Suspended steel wire mesh	9433LL Meal,Eberle Nafag AG,CH Lot no. 44/00	38	0	160	1
4	HSD/CPB-WU, Harlan Winkelmann GmbH, Germany	low-dust wood granules, Sniff: Typ BK 8/15	Eberle Nafag AG, CH Gossau, NAFAG Lot no.9349	31	0	163	3
5	Crj:CD(SD) IGS; Hino Breeding Ctr.	Sunflake®, Charles River, Japan	Oriental Yeast Co., Ltd, CRF-1 No lot number provided	42-44	100	231	3
6	Crl CD®(SD) IGS BR; Charles River, France	Autoclaved sawdust	AO4 C pelleted maintenance diet, batch No: 00331	42	100	254	3
7	CRL:CD(SD) IGS BR; Charles River, USA	Suspended steel wire mesh	PMI Certified 5002 Mash Lot no. May 22 00 3A	42	67	311	3
8	Crj:CD(SD)IGS; Charles River, Japan	Suspended steel wire mesh	Clea Japan Co., Ltd.; CE-2, Lot no. E2050-P8	40	100	213	1
9	CD Sprague-Dawley; Charles River, UK	Suspended steel wire mesh	Special Diet Services RM1(E) SQC expanded pellet (lot 6706)	42	100	237	3
10	Jcl:Wistar; Fuji Farm, Clea, Japan	Suspended steel wire mesh	Clea Japan, Ltd., CE-2, Lot no. E2050-P8	41-43	100	214	3
12	Crj CD(SD)IGS SPF/VAF; Charles River Japan	Suspended steel wire mesh	Oriental Yeast Co., Ltd., CRF-1, Lot no. 000405	41-44	100	256	1
13	Crj:CD(SD)IGS; Tsukuba Facility, Charles River Japan	Autoclaved hardwood chips (Beta Chip)	Oriental Yeast Co., Ltd., CRF-1, Lot no. 000412A1	44-46	100	265	2
14	Sprague-Dawley; Korea FDA	Autoclaved elm wood	PMI Lab Diet, 5014	40	83	224	3
15	Crj:CD(SD)IGS; Charles River, Japan	Autoclaved "White flake®"	Oriental Yeast Co., Ltd., CRF-1, Lot no. 00.05.09	42	100	223	3
16	CD; Charles River, Raleigh, NC, US	Sani-chips cage litter, P.J. Murphy	Pelleted Purina Certified Chow 5002 No lot number provided	44-46	83	349	1
17	Crj:CD(SD)IGS; Charles River, Japan	Beta chip, Northeastern Products, USA	Oriental Yeast Co., Ltd., CRF-1, Lot no. 000208, 000602	44	100	234	3

PPS: Preputial separation

Suppl. Material Table 2. Rat strains and suppliers, animal ages, and husbandry conditions used for the Flutamide dose response studies.

LAB	Rat Strain	Bedding material	Diet	Age at Castration (Days)	Age on Study (Days)	% Controls with PPS	Starting Body Wt (gms)	Rats per Cage
5	Crj:CD(SD) IGS	Sunflake®	Oriental Yeast Co., Ltd., CRF-1 Tokyo, Japan	42-44	51-53	100	229	3
8	Crj:CD(SD)IGS	Suspended steel wire mesh	Clea Japan Ltd., CE-2, Lot no. E2050-P8	40-42	46-48	100	209	1
10	Jcl:Wistar	Suspended steel wire mesh	Clea Japan Ltd., CE-2 Lot no. E2050-P8	41-43	47-49	NR.	230	3
12	Crj CD(SD)IGS SPF/VAF	Suspended steel wire mesh	Oriental Yeast Co., Ltd., CRF-1 Lot no. 000405	41-44	52-55	100	256	1
13	Crj:CD(SD)IGS	Autoclaved hardwood chips (Beta Chip)	Oriental Yeast Co., Ltd., CRF-1 Lot no. 000412 A1	44-46	51-53	100	255	2
15	Crj:CD(SD)IGS	Autoclaved "White flake®"	Oriental Yeast Co., Ltd., CRF-1 Lot no. 000509	41-43	48-50	NR	207	3
17	Crj:CD(SD)IGS	Beta chip	Oriental Yeast Co., Ltd., CRF-1 Lot no. 000208, 000602	43-45	50-52	100	231	2

PPS: Preputial separation
NR: not reported

Suppl. Material Table 3. Dose Response for Ventral Prostate in Phase-1A (mg, mean ± SD)

	Testosterone Propionate (mg/kg-bw/d)					
	**0**	**0.1**	**0.2**	**0.4**	**0.8**	**1.6**
Lab 1	23.0 ± 7.82	56.4 ± 12.07*,^	100.2 ± 15.10*,^	135.2 ± 8.61*,^	176.8 ± 32.61*,^	194.8 ± 40.85*,^
Lab 2	13.3 ± 2.97	50.4 ± 18.26*,^	101.7 ± 60.62*,^	146.6 ± 20.61*,^	228.4 ± 42.10*,^	249.1 ± 37.37*,^
Lab 3	12.3 ± 4.37	34.0 ± 7.80*,^	74.2 ± 17.51*,^	119.5 ± 21.95*,^	145.0 ± 12.36*,^	152.2 ± 23.89*,^
Lab 4	28.0 ± 10.26	39.2 ± 9.89*	64.2 ± 11.84*,^	96.0 ± 23.69*,^	149.3 ± 42.82*,^	185.7 ± 27.58*,^
Lab 5	16.3 ± 4.22	90.6 ± 25.67*,^	117.7 ± 23.91*,^	209.3 ± 23.90*,^	257.1 ± 27.41*,^	266.3 ± 60.63*,^
Lab 6	23.1 ± 7.69	83.5 ± 32.80*,^	80.2 ± 19.98*,^	141.6 ± 25.33*,^	257.0 ± 82.90*,^	344.0 ± 99.20*,^
Lab 7	34.5 ± 18.68	52.5 ± 20.25	76.1 ± 46.68*	117.1 ± 46.93*,^	167.9 ± 49.86*,^	206.5 ± 69.14*,^
Lab 8	17.4 ± 5.72	54.8 ± 11.87*,^	106.8 ± 22.77*,^	186.2 ± 16.98*,^	220.5 ± 28.80*,^	245.1 ± 31.52*,^
Lab 9	23.2 ± 10.82	95.9 ± 114.18*,^	111.4 ± 28.64*,^	193.1 ± 65.13*,^	225.8 ± 38.36*,^	223.1 ± 24.15*,^
Lab 10	11.1 ± 2.78	52.1 ± 8.41*,^	89.8 ± 24.13*,^	139.6 ± 25.68*,^	155.7 ± 30.92*,^	192.5 ± 34.45*,^
Lab 12	19.8 ± 5.66	72.3 ± 21.99*,^	129.8 ± 11.42*,^	176.4 ± 18.30*,^	271.5 ± 42.13*,^	292.2 ± 44.59*,^
Lab 13	26.8 ± 8.85	101.3 ± 16.01*,^	186.3 ± 10.59*,^	255.4 ± 60.45*,^	339.1 ± 56.56*,^	412.5 ± 99.72*,^
Lab 14	34.2 ± 8.17	68.9 ± 6.63*,^	104.6 ± 25.43*,^	179.5 ± 26.50*,^	245.0 ± 23.64*,^	310.7 ± 40.83*,^
Lab 15	19.2 ± 4.38	95.2 ± 23.83*,^	140.0 ± 14.45*,^	230.3 ± 33.82*,^	322.0 ± 50.01*,^	311.4 ± 41.47*,^
Lab 16	15.4 ± 5.34	76.7 ± 27.32*,^	115.4 ± 29.58*,^	183.1 ± 30.60*,^	263.8 ± 34.87*,^	270.8 ± 48.54*,^
Lab 17	29.9 ± 6.30	105.5 ± 10.05*,^	168.5 ± 44.01*,^	246.5 ± 34.91*,^	296.0 ± 40.56*,^	332.4 ± 67.71*,^
Avg. Wt. (mg) [CV]	21.7 [48]	70.6 *,^ [54]	110.4 *,^ [38]	172.2 *,^ [32]	232.6 *,^ [30]	261.8 *,^ [32]

* Significant using t-test group pairwise comparison ($p < 0.05$)
^ Significant using Dunnett's multiple comparison with either starting or terminal body weight adjustment ($p < 0.05$)

Suppl. Material Table 4. Dose Response for Seminal Vesicles and Coagulating Glands in Phase-1A (mg, mean ± SD)

	Testosterone Propionate (mg/kg-bw/d)					
	0	0.1	0.2	0.4	0.8	1.6
Lab 1	76.8 ± 6.05	150.5 ± 14.71*,^	266.1 ± 47.54*,^	397.4 ± 108.72*,^	616.8 ± 71.33*,^	922.0 ± 87.20*,^
Lab 2	67.2 ± 15.35	140.4 ± 40.11*,^	243.6 ± 113.62*,^	500.4 ± 225.59*,^	767.8 ± 159.49*,^	1050.0 ± 128.91*,^
Lab 3	15.3 ± 2.25	33.0 ± 10.08*,^	109.5 ± 24.84*,^	238.2 ± 58.61*,^	360.2 ± 52.47*,^	559.5 ± 54.72*,^
Lab 4	46.7 ± 8.71	129.7 ± 14.24*,^	235.2 ± 31.68*,^	356.3 ± 91.88*,^	577.3 ± 68.16*,^	704.7 ± 95.02*,^
Lab 5	51.5 ± 5.15	230.2 ± 62.88*,^	306.9 ± 159.98*,^	573.9 ± 79.88*,^	808.3 ± 66.13*,^	958.5 ± 230.14*,^
Lab 6	75.4 ± 13.62	170.6 ± 37.84*,^	245.1 ± 47.86*,^	547.7 ± 102.34*,^	827.0 ± 152.45*,^	1243.4 ± 261.82*,^
Lab 7	84.1 ± 36.20	117.1 ± 44.90	274.7 ± 121.28*,^	476.8 ± 114.65*,^	733.3 ± 74.03*,^	1026.8 ± 117.45*,^
Lab 8	47.8 ± 6.64	180.1 ± 58.68*,^	360.7 ± 82.07*,^	633.7 ± 109.80*,^	836.5 ± 178.85*,^	1180.2 ± 197.29*,^
Lab 9	65.5 ± 47.74	131.6 ± 31.65*,^	345.0 ± 81.42*,^	581.8 ± 215.71*,^	792.3 ± 312.29*,^	902.9 ± 174.91*,^
Lab 10	30.2 ± 5.57	119.1 ± 31.39*,^	270.2 ± 32.14*,^	395.5 ± 34.90*,^	614.9 ± 63.03*,^	886.3 ± 105.22*,^
Lab 12	39.4 ± 10.79	159.2 ± 32.04*,^	371.0 ± 63.59*,^	611.2 ± 120.70*,^	888.4 ± 111.22*,^	1211.9 ± 186.68*,^
Lab 13	65.7 ± 5.39	195.2 ± 75.34*,^	366.6 ± 52.22*,^	651.4 ± 130.02*,^	1034.6 ± 109.89*,^	1214.2 ± 133.99*,^
Lab 14	41.7 ± 10.37	118.8 ± 31.10*,^	261.3 ± 66.50*,^	524.0 ± 42.84*,^	646.5 ± 134.70*,^	1103.8 ± 201.21*,^
Lab 15	38.4 ± 9.74	176.0 ± 24.33*,^	360.7 ± 56.74*,^	647.6 ± 114.79*,^	970.1 ± 116.53*,^	1110.8 ± 97.99*,^
Lab 16	47.7 ± 6.09	193.9 ± 55.02*,^	375.2 ± 34.76*,^	552.6 ± 81.74*,^	936.5 ± 94.33*,^	1169.8 ± 92.70*,^
Lab 17	58.7 ± 7.92	191.0 ± 37.11*,^	374.2 ± 63.81*,^	506.9 ± 52.70*,^	946.1 ± 67.02*,^	1226.1 ± 196.34*,^
Avg. Wt. (mg) [CV]	53.2 [45]	152.3 *,^ [39]	297.9 *,^ [32]	512.2 *,^ [30]	772.3 *,^ [21]	1029.4 *,^ [25]

* Significant using t-test group pairwise comparison ($p < 0.05$)

^ Significant using Dunnett's multiple comparison with either starting or terminal body weight adjustment ($p < 0.05$)

Suppl. Material Table 5. Dose Response for Levator Ani and Bulbocavernosus in Phase-1A (mg, mean ± SD)

	Testosterone Propionate (mg/kg-bw/d)					
	0	0.1	0.2	0.4	0.8	1.6
Lab 1	86.3 ± 13.14	143.5 ± 19.20[*,^]	195.9 ± 22.27[*,^]	240.4 ± 20.71[*,^]	284.7 ± 21.17[*,^]	340.5 ± 44.02[*,^]
Lab 2	113.6 ± 17.55	192.1 ± 40.14[*,#]	277.9 ± 95.67[*,^]	379.5 ± 88.26[*,^]	421.3 ± 25.18[*,^]	463.0 ± 47.21[*,^]
Lab 3	119.5 ± 13.03	177.7 ± 38.80[*,^]	243.3 ± 34.06[*,^]	321.7 ± 32.89[*,^]	357.3 ± 35.51[*,^]	420.3 ± 55.51[*,^]
Lab 4	65.9 ± 21.56	146.1 ± 36.01[*,^]	182.8 ± 35.93[*,^]	227.1 ± 47.09[*,^]	247.1 ± 72.02[*,^]	314.4 ± 42.44[*,^]
Lab 5	200.2 ± 18.29	382.4 ± 54.05[*,^]	514.1 ± 38.55[*,^]	660.0 ± 52.80[*,^]	755.1 ± 28.12[*,^]	734.2 ± 42.43[*,^]
Lab 6	287.5 ± 48.51	406.7 ± 55.95[*,^]	465.1 ± 47.08[*,^]	615.9 ± 55.00[*,^]	812.1 ± 81.11[*,^]	889.6 ± 115.11[*,^]
Lab 7	169.0 ± 81.17	278.0 ± 41.79[*,^]	352.4 ± 73.84[*,^]	543.6 ± 83.75[*,^]	593.6 ± 100.69[*,^]	645.7 ± 140.90[*,^]
Lab 8	205.4 ± 20.18	382.0 ± 22.64[*,^]	509.2 ± 40.29[*,^]	638.5 ± 52.14[*,^]	743.8 ± 66.84[*,^]	748.8 ± 72.17[*,^]
Lab 9	173.5 ± 41.54	245.2 ± 99.07[*]	421.5 ± 31.55[*,^]	535.7 ± 29.30[*,^]	596.1 ± 76.58[*,^]	590.1 ± 64.25[*,^]
Lab 10	174.0 ± 25.45	337.6 ± 32.43[*,^]	450.7 ± 37.43[*,^]	543.3 ± 33.45[*,^]	611.8 ± 63.00[*,^]	708.9 ± 36.86[*,^]
Lab 12	176.0 ± 14.45	340.5 ± 70.11[*,^]	515.7 ± 49.61[*,^]	574.1 ± 53.41[*,^]	729.7 ± 89.41[*,^]	837.8 ± 41.05[*,^]
Lab 13	260.2 ± 30.32	477.1 ± 71.23[*,^]	599.7 ± 47.54[*,^]	832.0 ± 90.84[*,^]	901.2 ± 101.56[*,^]	991.0 ± 50.67[*,^]
Lab 14	194.4 ± 35.96	350.8 ± 69.58[*,^]	397.1 ± 48.81[*,^]	643.8 ± 18.47[*,^]	661.2 ± 52.50[*,^]	830.2 ± 62.72[*,^]
Lab 15	201.8 ± 26.55	400.4 ± 40.69[*,^]	505.6 ± 54.97[*,^]	610.9 ± 50.30[*,^]	704.9 ± 63.75[*,^]	743.6 ± 54.47[*,^]
Lab 16	212.9 ± 25.26	436.8 ± 50.49[*,^]	523.2 ± 34.42[*,^]	621.1 ± 56.83[*,^]	719.5 ± 82.21[*,^]	790.4 ± 65.73[*,^]
Lab 17	253.5 ± 35.00	413.2 ± 66.44[*,^]	585.6 ± 44.48[*,^]	691.3 ± 46.20[*,^]	805.7 ± 56.80[*,^]	910.0 ± 109.62[*,^]
Avg. Wt. (mg) [CV] [a]	96.3 [28]	164.8 [*,^] [23]	225.0 [*,^] [29]	292.2 [*,^] [28]	327.6 [*,^] [24]	384.6 [*,^] [20]
Avg. Wt. (mg) [CV] [b]	209.0 [24]	370.9 [*,^] [22]	486.7 [*,^] [18]	625.8 [*,^] [16]	719.6 [*,^] [15]	785.0 [*,^] [17]

* Significant using t-test group pairwise comparison ($p < 0.05$)
# Significant using Dunnett's multiple comparisons with starting body weight adjustment only ($p < 0.05$)
^ Significant using Dunnett's multiple comparisons with either starting or terminal body weight adjustment ($p < 0.05$)
[a] Average weight and CV for labs 1-4, which dissected and weighted only the levator ani muscle and not the bulbocavernosus muscle.
[b] Average weight and CV for labs 5-17, which dissected and weighed both the levator ani and bulbocavernosus muscles.

Suppl. Material Table 6. Dose Response for Glans Penis in Phase-1A (mg, mean ± SD)

	Testosterone Propionate (mg/kg-bw/d)					
	0	0.1	0.2	0.4	0.8	1.6
Lab 1	50.1 ± 3.36	71.4 ± 4.74*,^	76.5 ± 6.73*,^	87.4 ± 7.23*,^	91.8 ± 10.73*,^	88.6 ± 4.87*,^
Lab 2	40.8 ± 8.75	62.5 ± 13.74*	69.0 ± 16.32*,^	67.7 ± 16.01*,^	62.8 ± 15.96*,^	76.4 ± 19.87*,^
Lab 3	28.3 ± 6.09	42.3 ± 12.50*,^	49.3 ± 10.69*,^	57.7 ± 5.35*,^	61.8 ± 3.71*,^	63.0 ± 6.10*,^
Lab 4	45.2 ± 19.26	68.9 ± 33.99	73.3 ± 9.36*,#	72.9 ± 12.77*	74.0 ± 11.75*,#	85.6 ± 9.12*,^
Lab 5	53.5 ± 6.50	79.5 ± 3.25*,^	78.9 ± 5.01*,^	95.0 ± 12.76*,^	90.8 ± 7.38*,^	96.6 ± 7.16*,^
Lab 6	58.8 ± 11.25	73.7 ± 6.23*,^	80.8 ± 12.43*,^	98.9 ± 8.46*,^	101.7 ± 3.05*,^	106.4 ± 12.98*,^
Lab 7	49.2 ± 14.76	72.5 ± 11.48*,^	70.9 ± 6.11*,^	93.4 ± 14.24*,^	92.0 ± 22.01*,^	93.8 ± 15.06*,^
Lab 8	48.1 ± 2.33	69.5 ± 2.78*,^	75.1 ± 4.18*,^	81.8 ± 2.43*,^	79.0 ± 5.60*,^	83.2 ± 6.14*,^
Lab 9	46.7 ± 6.58	66.1 ± 7.71*,^	78.4 ± 13.23*,^	78.0 ± 9.38*,^	84.3 ± 5.43*,^	86.4 ± 8.62*,^
Lab 10	30.9 ± 3.80	52.4 ± 10.86*,^	73.6 ± 6.48*,^	82.0 ± 7.00*,^	85.2 ± 8.66*,^	88.2 ± 8.42*,^
Lab 12	49.0 ± 2.70	69.5 ± 10.70*,^	86.2 ± 9.46*,^	79.2 ± 6.79*,^	88.3 ± 13.74*,^	96.3 ± 13.44*,^
Lab 13	51.6 ± 3.23	71.4 ± 10.18*,^	85.3 ± 8.20*,^	92.0 ± 12.70*,^	103.6 ± 10.10*,^	107.8 ± 16.06*,^
Lab 14	35.2 ± 2.51	60.6 ± 6.57*,^	67.9 ± 3.08*,^	83.2 ± 12.92*,^	80.8 ± 10.99*,^	89.0 ± 14.20*,^
Lab 15	65.9 ± 8.69	100.4 ± 8.33*,^	110.8 ± 15.07*,^	115.7 ± 12.54*,^	133.1 ± 11.82*,^	118.4 ± 14.66*,^
Lab 16	49.8 ± 9.12	74.3 ± 7.33*,^	78.8 ± 6.52*,^	95.2 ± 8.30*,^	96.2 ± 6.54*,^	95.6 ± 7.81*,^
Lab 17	66.1 ± 8.46	85.1 ± 10.74*,^	87.2 ± 6.86*,^	93.8 ± 4.27*,^	97.7 ± 8.90*,^	107.7 ± 10.59*,^
Avg. Wt. (mg) [CV]	48.1 [27]	70.0 *,^ [24]	77.6 *,^ [19]	85.8 *,^ [19]	88.9 *,^ [22]	92.7 *,^ [18]

* Significant using t-test group pairwise comparison ($p < 0.05$).
# Significant using Dunnett's multiple comparisons with starting body weight adjustment only ($p < 0.05$).
^ Significant using Dunnett's multiple comparisons with either starting or terminal body weight adjustment ($p < 0.05$).

Suppl. Material Table 7. Dose Response for Cowper's Glands in Phase-1A (mg, mean ± SD)

	Testosterone Propionate (mg/kg-bw/d)					
	0	0.1	0.2	0.4	0.8	1.6
Lab 1	7.0 ± 1.66	14.7 ± 1.26[*,^]	24.2 ± 3.54[*,^]	36.3 ± 4.70[*,^]	49.1 ± 8.05[*,^]	56.0 ± 3.92[*,^]
Lab 2	3.8 ± 1.13	10.3 ± 5.36[*,#]	21.2 ± 5.48[*,^]	32.7 ± 8.68[*,^]	49.7 ± 6.05[*,^]	51.8 ± 13.97[*,^]
Lab 3	2.5 ± 0.55	8.3 ± 2.58[*,^]	16.2 ± 2.99[*,^]	23.0 ± 2.00[*,^]	30.0 ± 3.79[*,^]	33.0 ± 3.74[*,^]
Lab 4	1.6 ± 1.53	7.2 ± 1.97[*,^]	12.1 ± 2.55[*,^]	16.2 ± 3.76[*,^]	26.4 ± 2.84[*,^]	28.5 ± 2.84[*,^]
Lab 5	6.1 ± 2.80	22.7 ± 4.60[*,^]	32.2 ± 9.39[*,^]	42.0 ± 6.09[*,^]	56.1 ± 9.75[*,^]	63.0 ± 16.21[*,^]
Lab 6	10.3 ± 2.41	27.9 ± 7.62[*,^]	29.4 ± 4.84[*,^]	44.1 ± 4.48[*,^]	57.7 ± 13.05[*,^]	75.8 ± 4.22[*,^]
Lab 7	10.8 ± 6.38	16.5 ± 7.13	21.2 ± 9.72*	35.5 ± 8.79[*,^]	39.7 ± 18.99[*,^]	55.4 ± 12.76[*,^]
Lab 8	6.6 ± 2.05	15.9 ± 3.04[*,^]	28.6 ± 6.74[*,^]	35.6 ± 5.30[*,^]	44.7 ± 10.44[*,^]	54.0 ± 13.95[*,^]
Lab 9	5.6 ± 3.58	15.2 ± 2.29[*,^]	24.1 ± 6.15[*,^]	31.2 ± 7.45[*,^]	43.4 ± 6.61[*,^]	47.3 ± 5.51[*,^]
Lab 10	6.4 ± 0.97	18.5 ± 2.43[*,^]	29.3 ± 3.98[*,^]	37.2 ± 6.60[*,^]	43.2 ± 5.49[*,^]	52.1 ± 3.47[*,^]
Lab 12	5.9 ± 2.68	16.2 ± 4.69[*,^]	29.7 ± 4.19[*,^]	37.6 ± 3.67[*,^]	51.1 ± 13.36[*,^]	66.7 ± 13.89[*,^]
Lab 13	11.1 ± 3.71	25.6 ± 2.63[*,^]	38.5 ± 7.58[*,^]	52.5 ± 8.51[*,^]	71.6 ± 14.40[*,^]	71.1 ± 10.35[*,^]
Lab 14	4.5 ± 1.05	11.9 ± 1.72[*,^]	25.3 ± 6.03[*,^]	33.3 ± 6.69[*,^]	39.7 ± 5.76[*,^]	66.4 ± 11.36[*,^]
Lab 15	5.8 ± 1.85	25.5 ± 9.78[*,^]	31.5 ± 4.80[*,^]	45.4 ± 8.47[*,^]	63.7 ± 8.62[*,^]	71.3 ± 14.43[*,^]
Lab 16	5.5 ± 0.75	19.8 ± 5.68[*,^]	28.3 ± 3.03[*,^]	46.1 ± 9.24[*,^]	56.3 ± 9.70[*,^]	58.6 ± 11.70[*,^]
Lab 17	15.6 ± 8.68	31.1 ± 6.10[*,^]	45.0 ± 9.07[*,^]	53.6 ± 6.47[*,^]	68.0 ± 10.31[*,^]	73.1 ± 3.32[*,^]
Avg. Wt. (mg) [CV]	6.8 [68]	17.9 [*,^] [45]	27.3 [*,^] [35]	37.6 [*,^] [30]	49.4 [,^] [31]	57.7 [*,^] [28]

* Significant using t-test group pairwise comparison ($p < 0.05$).
# Significant using Dunnett's multiple comparisons with starting body weight adjustment only ($p < 0.05$).
^ Significant using Dunnett's multiple comparisons with either starting or terminal body weight adjustment ($p < 0.05$).

Suppl. Material Table 8. Dose Response for Ventral Prostate in Phase-1B (mg, mean ± SD)

Testosterone Propionate	0	0.2 mg/kg-bw/day					
Flutamide (mg/kg-bw/d)	0	0	0.1	0.3	1	3	10
Lab 5	16.1 ± 3.76 [a]	114.4 ± 10.60	117.0 ± 10.43	102.9 ± 18.21	63.7 ± 18.21[*,^]	31.2 ± 5.29[*,^]	19.4 ± 2.33[*,^]
Lab 8	14.6 ± 5.43	105.4 ± 34.07	105.6 ± 35.04	81.2 ± 19.53	54.2 ± 19.53[*,^]	27.9 ± 9.50[*,^]	23.1 ± 9.75[*,^]
Lab 10	not done	not done	not done	not done	not done	not done	not done
Lab 12	19.0 ± 8.19 [a]	127.6 ± 13.60	107.1 ± 23.52	101.2 ± 18.84[*]	56.3 ± 18.84[*,^]	31.6 ± 5.29[*,^]	22.3 ± 3.32[*,^]
Lab 13	23.5 ± 6.76	141.9 ± 27.89	149.5 ± 30.62	99.7 ± 17.02[*,#]	85.2 ± 17.02[*,^]	48.0 ± 7.70[*,^]	31.9 ± 5.80[*,^]
Lab 15	22.3 ± 4.12 [a]	131.5 ± 16.86	150.4 ± 41.79	108.5 ± 18.34	83.3 ± 18.34[*,^]	38.4 ± 4.65[*,^]	25.3 ± 1.72[*,^]
Lab 17	15.1 ± 4.76	139.7 ± 25.09	134.2 ± 19.47	116.9 ± 35.65	69.0 ± 35.65[*,^]	32.9 ± 9.96[*,^]	26.3 ± 4.26[*,^]
Avg. Wt. (mg) [CV]	18.4 [a] [39]	126.7 [20]	127.3 [26]	101.7 [*,^] [23]	68.6 [*,^] [30]	35.0 [*,^] [27]	24.7 [*,^] [25]
Testosterone Propionate	0	0.4 mg/kg-bw/day					
Flutamide (mg/kg-bw/d)	0	0	0.1	0.3	1	3	10
Lab 5	16.1 ± 3.76 [a]	211.0 ± 35.00	177.5 ± 35.94	174.7 ± 22.60	116.9 ± 21.66[*,^]	59.2 ± 21.66[*,^]	28.0 ± 4.58[*,^]
Lab 8	not done	not done	not done	not done	not done	not done	not done
Lab 10	9.4 ± 2.45	162.7 ± 29.74	128.5 ± 23.96[*]	104.1 ± 16.99[*,^]	67.1 ± 16.99[*,^]	37.2 ± 5.20[*,^]	19.9 ± 3.00[*,^]
Lab 12	19.0 ± 8.19 [a]	213.6 ± 21.51	171.1 ± 46.11	173.9 ± 24.29	112.7 ± 32.91[*,^]	52.0 ± 32.91[*,^]	25.5 ± 9.08[*,^]
Lab 13	25.7 ± 5.56	233.5 ± 47.72	228.0 ± 46.64	196.8 ± 23.37	175.6 ± 23.37[*]	79.8 ± 13.65[*,^]	45.5 ± 12.88[*,^]
Lab 15	22.3 ± 4.12 [a]	268.2 ± 58.43	212.6 ± 33.02[*]	202.3 ± 20.10[*,^]	136.3 ± 12.98[*,^]	67.7 ± 12.98[*,^]	30.9 ± 2.40[*,^]
Lab 17	not done	not done	not done	not done	not done	not done	not done
Avg. Wt. (mg) [CV]		217.8 [24]	183.5 [*] [27]	170.4 [*,^] [24]	121.7 [*,^] [34]	59.2 [*,^] [35]	30.0 [*,^] [37]

* Significant using t-test group pairwise comparison ($p < 0.05$).
# Significant using Dunnett's multiple comparisons with starting body weight adjustment only ($p < 0.05$).
^ Significant using Dunnett's multiple comparison with either starting or terminal body weight adjustment ($p < 0.05$).
[a] A single vehicle control was used because the 0.2 and the 0.4 mg/kg-bw/d TP series were run concurrently. Therefore, only one average weight and CV which incorporates all vehicle controls are reported.

Suppl. Material Table 9. Dose Response for Seminal Vesicles and Coagulating Glands in Phase-1B (mg, mean ± SD)

Testosterone Propionate	0	0.2 mg/kg-bw/day					
Flutamide (mg/kg-bw/d)	0	0	0.1	0.3	1	3	10
Lab 5	36.7 ± 9.12 [a]	314.7 ± 41.40	251.7 ± 26.89[*]	204.2 ± 46.36[*,^]	123.2 ± 22.26[*,^]	53.9 ± 11.96[*,^]	40.9 ± 6.19[*,^]
Lab 8	32.0 ± 6.35	348.4 ± 75.13	263.5 ± 87.97	247.3 ± 82.73[*]	116.9 ± 37.09[*,^]	60.5 ± 21.18[*,^]	44.6 ± 6.97[*,^]
Lab 10	not done	not done	not done	not done	not done	not done	not done
Lab 12	53.0 ± 15.09 [a]	368.5 ± 88.42	366.4 ± 73.04	247.9 ± 66.27[*,^]	137.4 ± 51.52[*,^]	70.7 ± 8.16[*,^]	61.6 ± 13.49[*,^]
Lab 13	69.6 ± 7.80	315.9 ± 64.85	288.5 ± 79.60	246.7 ± 70.45	137.1 ± 34.62[*,^]	86.3 ± 17.11[*,^]	74.3 ± 7.91[*,^]
Lab 15	46.6 ± 8.22 [a]	288.6 ± 82.17	232.5 ± 75.51	205.8 ± 37.34[*]	116.5 ± 32.39[*,^]	67.8 ± 17.32[*,^]	50.0 ± 4.69[*,^]
Lab 17	51.0 ± 10.99	440.2 ± 97.50	320.2 ± 49.06[*]	299.5 ± 85.06[*,^]	166.6 ± 66.55[*,^]	83.1 ± 17.30[*,^]	66.6 ± 6.63[*,^]
Avg. Wt. (mg) [CV]	47.2 [a] [34]	346.0 [25]	287.1 [*,^] [27]	241.9 [*,^] [29]	132.9 [*,^] [33]	70.3 [*,^] [27]	56.3 [*,^] [25]
Testosterone Propionate	0	0.4 mg/kg-bw/day					
Flutamide (mg/kg-bw/d)	0	0	0.1	0.3	1	3	10
Lab 5	36.7 ± 9.12 [a]	539.1 ± 64.92	450.4 ± 78.22	409.8 ± 51.70[*]	243.0 ± 53.62[*,^]	108.5 ± 27.91[*,^]	44.5 ± 7.41[*,^]
Lab 8	not done	not done	not done	not done	not done	not done	not done
Lab 10	30.5 ± 5.28	507.1 ± 36.80	418.5 ± 94.47[*]	352.9 ± 48.06[*,^]	189.3 ± 46.92[*,^]	83.1 ± 13.41[*,^]	40.8 ± 6.18[*,^]
Lab 12	53.0 ± 15.09 [a]	588.4 ± 118.74	492.9 ± 103.11	495.4 ± 139.72	294.6 ± 66.11[*,^]	122.3 ± 39.28[*,^]	64.7 ± 7.75[*,^]
Lab 13	63.8 ± 9.57	673.7 ± 154.53	646.3 ± 81.72	572.6 ± 122.56	402.2 ± 97.62[*,^]	167.6 ± 58.96[*,^]	82.5 ± 13.98[*,^]
Lab 15	46.6 ± 8.22 [a]	592.1 ± 63.17	431.2 ± 39.11[*,^]	406.4 ± 65.27[*,^]	266.1 ± 36.65[*,^]	105.9 ± 27.96[*,^]	60.8 ± 9.66[*,^]
Lab 17	not done	not done	not done	not done	not done	not done	not done
Avg. Wt. (mg) [CV]		580.1 [*,^] [18]	487.9 [*,^] [23]	447.4 [*,^] [24]	279.0 [*,^] [33]	117.5 [*,^] [38]	58.7 [*,^] [30]

* Significant using t-test group pairwise comparison ($p < 0.05$).
^ Significant using Dunnett's multiple comparison with either starting or terminal body weight adjustment ($p < 0.05$).
[a] A single vehicle control was used because the 0.2 and the 0.4 mg/kg-bw/d TP series were run concurrently. Therefore, only one average weight and CV which incorporates all vehicle controls are reported.

Suppl. Material Table 10. Dose Response for Levator Ani and Bulbocavernosus Muscles in Phase-1B (mg, mean ± SD)

Testosterone Propionate	0	0.2 mg/kg-bw/day					
Flutamide (mg/kg-bw/d)	0	0	0.1	0.3	1	3	10
Lab 5	177.5 ± 23.47 [a]	444.2 ± 22.00	413.1 ± 26.89	387.0 ± 38.49*	305.6 ± 30.80*,^	238.6 ± 27.79*,^	188.7 ± 16.74*,^
Lab 8	175.2 ± 13.58	488.1 ± 25.66	493.4 ± 47.84	422.0 ± 60.67	358.8 ± 56.94*,^	243.0 ± 39.24*,^	213.9 ± 29.34*,^
Lab 10	not done	not done	not done	not done	not done	not done	not done
Lab 12	187.0 ± 19.82 [a]	482.7 ± 51.48	461.2 ± 83.50	412.3 ± 43.37	272.9 ± 37.96*,^	223.5 ± 21.46*,^	216.6 ± 39.04*,^
Lab 13	261.0 ± 26.50	514.5 ± 50.39	515.6 ± 66.47	440.6 ± 103.93	414.2 ± 69.32*,#	315.5 ± 47.88*,^	267.6 ± 20.87*,^
Lab 15	208.0 ± 30.78 [a]	442.4 ± 46.18	454.2 ± 52.93	415.2 ± 37.75	331.7 ± 35.02*,^	259.1 ± 30.32*,^	219.6 ± 19.19*,^
Lab 17	248.9 ± 34.93	565.3 ± 49.73	511.0 ± 84.35	514.0 ± 66.63	419.9 ± 75.41*,^	280.3 ± 35.79*,^	268.1 ± 29.19*,^
Avg. Wt. (mg) [CV]	206.2 [a] [22]	489.5 [12]	474.7 [15]	431.9 *,^ [16]	350.5 *,^ [21]	260.0 *,^ [17]	229.1 *,^ [17]
Testosterone Propionate	0	0.4 mg/kg-bw/day					
Flutamide (mg/kg-bw/d)	0	0	0.1	0.3	1	3	10
Lab 5	177.5 ± 23.47 [a]	577.9 ± 47.18	555.3 ± 64.88	547.2 ± 19.37	419.0 ± 68.81*,^	314.4 ± 36.61*,^	201.6 ± 35.00*,^
Lab 8	not done	not done	not done	not done	not done	not done	not done
Lab 10	166.8 ± 29.77	603.2 ± 60.34	595.9 ± 45.85	497.9 ± 28.21*,^	435.2 ± 32.38*,^	328.1 ± 36.51*,^	207.2 ± 22.39*,^
Lab 12	187.0 ± 19.82 [a]	605.8 ± 52.74	584.5 ± 54.65	549.1 ± 39.69	405.7 ± 57.07*,^	265.3 ± 35.76*,^	234.9 ± 47.56*,^
Lab 13	275.4 ± 28.67	783.8 ± 89.63	709.9 ± 83.94	659.1 ± 44.34*,^	576.8 ± 44.01*,^	390.8 ± 47.25*,^	321.3 ± 12.70*,^
Lab 15	208.0 ± 30.78 [a]	654.3 ± 63.39	587.6 ± 39.12	511.8 ± 51.33*,^	445.4 ± 48.32*,^	337.3 ± 46.15*,^	251.7 ± 19.35*,^
Lab 17	not done	not done	not done	not done	not done	not done	not done
Avg. Wt. (mg) [CV]		645.0 [15]	606.6 * [13]	553.0 *,^ [12]	456.4 *,^ [17]	327.2 *,^ [17]	243.3 *,^ [21]

* Significant using t-test group pairwise comparison ($p < 0.05$).
# Significant using Dunnett's multiple comparisons with starting body weight adjustment only ($p < 0.05$).
^ Significant using Dunnett's multiple comparison with either starting or terminal body weight adjustment ($p < 0.05$).
[a] A single vehicle control was used because the 0.2 and the 0.4 mg/kg-bw/d TP series were run concurrently. Therefore, only one average weight and CV which incorporates all vehicle controls are reported.

Suppl. Material Table 11. Dose Response for Glans Penis in Phase-1B (mg, mean ± SD)

Testosterone Propionate	0	0.2 mg/kg-bw/day					
Flutamide (mg/kg-bw/d)	0	0	0.1	0.3	1	3	10
Lab 5	48.6 ± 5.04 [a]	77.0 ± 4.36	78.4 ± 2.95	75.8 ± 3.50	67.6 ± 4.52[*,^]	57.7 ± 7.10[*,^]	51.3 ± 2.89[*,^]
Lab 8	40.1 ± 5.93	68.7 ± 9.13	70.8 ± 11.46	66.6 ± 5.27	66.1 ± 8.23	51.5 ± 5.81[*,^]	49.6 ± 5.45[*,^]
Lab 10	not done	not done	not done	not done	not done	not done	not done
Lab 12	55.1 ± 2.60 [a]	84.9 ± 6.85	80.7 ± 6.13	80.0 ± 5.75	68.8 ± 2.78[*,^]	60.6 ± 6.88[*,^]	56.3 ± 3.22[*,^]
Lab 13	49.3 ± 5.44	80.3 ± 5.31	76.5 ± 2.49	77.1 ± 9.99	70.1 ± 6.28[*]	59.1 ± 6.46[*,^]	58.7 ± 10.79[*,^]
Lab 15	64.8 ± 8.13 [a]	110.3 ± 12.96	92.4 ± 10.96[*]	93.0 ± 6.58[*,^]	86.8 ± 10.03[*,^]	73.9 ± 9.86[*,^]	62.9 ± 6.36[*,^]
Lab 17	62.5 ± 13.19	103.0 ± 9.04	91.2 ± 7.40[##]	87.4 ± 17.48[*,##]	82.9 ± 7.43[*,^]	73.7 ± 11.30[*,^]	72.7 ± 10.45[*,^]
Avg. Wt. (mg) [CV]	51.4 [a] [25]	87.4 [19]	81.7 [*] [13]	80.1 [*] [15]	73.7 [*,^] [14]	62.7 [*,^] [18]	58.6 [*,^] [18]
Testosterone Propionate	0	0.4 mg/kg-bw/day					
Flutamide (mg/kg-bw/d)	0	0	0.1	0.3	1	3	10
Lab 5	48.6 ± 5.04 [a]	82.7 ± 6.75	87.9 ± 2.87	81.0 ± 4.63	77.4 ± 6.56	70.0 ± 5.13[*,^]	56.6 ± 2.19[*,^]
Lab 8	not done	not done	not done	not done	not done	not done	not done
Lab 10	30.8 ± 4.56	80.9 ± 4.43	84.7 ± 6.37	75.9 ± 4.06	64.6 ± 4.40[*,^]	51.9 ± 8.64[*,^]	39.5 ± 7.20[*,^]
Lab 12	55.1 ± 2.60 [a]	90.8 ± 5.37	87.9 ± 4.36	88.9 ± 2.85	81.5 ± 3.95[*,^]	69.2 ± 4.57[*,^]	59.9 ± 1.55[*,^]
Lab 13	48.1 ± 1.44	95.8 ± 8.49	93.5 ± 8.34	88.6 ± 7.54	82.9 ± 11.22[*,#]	67.4 ± 5.01[*,^]	56.8 ± 4.70[*,^]
Lab 15	64.8 ± 8.13 [a]	114.7 ± 4.78	118.1 ± 14.22	106.9 ± 12.70	102.9 ± 10.91	87.2 ± 12.94[*,^]	77.4 ± 7.28[*,^]
Lab 17	not done	not done	not done	not done	not done	not done	not done
Avg. Wt. (mg) [CV]		93.0 [15]	94.4 [15]	88.3 [*] [14]	81.8 [*,##] [18]	69.1 [*,^] [20]	58.0 [*,^] [23]

* Significant using t-test group pairwise comparison ($p < 0.05$).
# Significant using Dunnett's multiple comparisons with starting body weight adjustment only ($p < 0.05$).
Significant using Dunnett's multiple comparisons with terminal body weight adjustment only ($p < 0.05$).
^ Significant using Dunnett's multiple comparison with either starting or terminal body weight adjustment ($p < 0.05$).
[a] A single vehicle control was used because the 0.2 and the 0.4 mg/kg-bw/d TP series were run concurrently. Therefore, only one average weight and CV which incorporates all vehicle controls are reported.

Suppl. Material Table 12. Dose Response for Cowper's Glands in Phase-1B (mg, mean ± SD)

Testosterone Propionate	0	0.2 mg/kg-bw/day					
Flutamide (mg/kg-bw/d)	0	0	0.1	0.3	1	3	10
Lab 5	5.0 ± 1.49 [a]	25.5 ± 7.04	27.3 ± 6.09	20.5 ± 3.64	17.2 ± 4.71*,^	9.8 ± 2.91*,^	6.8 ± 0.73*,^
Lab 8	6.4 ± 2.74	25.1 ± 3.80	24.7 ± 4.52	20.6 ± 3.15	14.5 ± 2.51*,^	9.2 ± 2.03*,^	7.0 ± 1.84*,^
Lab 10	not done	not done	not done	not done	not done	not done	not done
Lab 12	9.3 ± 1.14 [a]	32.1 ± 4.96	30.7 ± 3.04	24.4 ± 4.63*,^	18.1 ± 3.05*,^	11.7 ± 3.41*,^	8.8 ± 1.13*,^
Lab 13	9.9 ± 2.03	31.1 ± 3.07	31.1 ± 4.68	24.2 ± 6.79*	23.1 ± 4.86*	13.9 ± 4.81*,^	12.2 ± 2.38*,^
Lab 15	8.4 ± 1.14 [a]	26.7 ± 3.51	26.6 ± 8.08	20.0 ± 4.90*	21.6 ± 3.24	10.7 ± 3.08*,^	7.2 ± 1.59*,^
Lab 17	10.0 ± 3.41	37.9 ± 11.37	30.7 ± 3.04	27.2 ± 6.43	25.7 ± 6.48	13.9 ± 4.31*,^	11.0 ± 3.48*,^
Avg. Wt. (mg) [CV]	7.9 [a] [36]	29.7 [24]	28.5 [29]	22.8 *,^ [24]	20.0 *,^ [28]	11.5 *,^ [33]	8.8 *,^ [32]
Testosterone Propionate	0	0.4 mg/kg-bw/day					
Flutamide (mg/kg-bw/d)	0	0	0.1	0.3	1	3	10
Lab 5	5.0 ± 1.49 [a]	40.0 ± 5.59	34.7 ± 5.52	33.8 ± 4.29	23.6 ± 3.72*,^	17.5 ± 7.11*,^	8.3 ± 1.72*,^
Lab 8	not done	not done	not done	not done	not done	not done	not done
Lab 10	5.3 ± 0.98	41.9 ± 2.17	39.2 ± 4.03	34.1 ± 4.22*,^	26.0 ± 2.47*,^	16.4 ± 4.41*,^	7.3 ± 2.04*,^
Lab 12	9.3 ± 1.14 [a]	45.4 ± 3.94	38.9 ± 8.25	33.9 ± 4.52*,^	26.0 ± 3.84*,^	15.6 ± 2.00*,^	11.9 ± 2.36*,^
Lab 13	10.4 ± 3.42	51.8 ± 9.70	52.1 ± 7.84	41.2 ± 3.88	38.3 ± 7.31*,^	24.7 ± 4.46*,^	15.4 ± 2.76*,^
Lab 15	8.4 ± 1.14 [a]	43.8 ± 5.53	38.4 ± 7.12	35.8 ± 4.75	30.2 ± 3.86*,^	16.6 ± 3.96*,^	9.1 ± 2.35*,^
Lab 17	not done	not done	not done	not done	not done	not done	not done
Avg. Wt. (mg) [CV]		44.6 [16]	40.6 * [21]	35.7 *,^ [14]	28.8 *,^ [24]	18.2 *,^ 30]	10.4 *,^ [35]

* Significant using t-test group pairwise comparison ($p < 0.05$).
^ Significant using Dunnett's multiple comparison with either starting or terminal body weight adjustment ($p < 0.05$).
[a] A single vehicle control was used because the 0.2 and the 0.4 mg/kg-bw/d TP series were run concurrently. Therefore, only one average weight and CV which incorporates all vehicle controls are reported.

Suppl. Material Table 13. Evaluation of statistical transformations used to normalise the Phase-1A data

Tissue	Laboratories	Most Appropriate Transformation
VP	1, 4, 14, 15	Log_{10}
	2, 3, 7, 8, 17	Untransformed
	5, 6, 10, 12, 16	Square root
	9, 13	No obvious transformation
SVCG	1, 3, 4, 5, 7, 8, 9,10, 14, 15	Log_{10}
	2, 6, 12, 13, 16, 17	Square root
LABC	1, 3, 4, 10, 12, 13	Log_{10}
	6, 7, 16, 17	Untransformed
	8, 15	Square root
	2, 5, 9,14	No obvious transformation
Glans penis	1, 9, 13, 15	Log_{10}
	2, 3, 5, 6, 7, 14, 16, 17	Untransformed
	8, 10, 12	Square root
	4	No obvious transformation
COWS	1,8, 12,13, 16	Log_{10}
	2, 5, 7, 9, 15, 17	Untransformed
	3, 4, 6, 10, 14	Square root

VP, Ventral Prostate; SVCG, paired seminal vesicles and coagulating glands; LABC, levator ani and bulbocavernosus muscles; COWS, Cowper's glands.

Suppl. Material Table 14. LOEL changes in Phase-1A as an effect of data transformation used

Tissue	Lab	LOEL (mg TP/kg-bw/d) with Log_{10} transformation	Most appropriate transformation	LOEL (mg TP/kg-bw/d) with most appropriate transformation
SVCG	6	0.1	Untransformed	0.4
LABC	2	0.1	Untransformed	0.2
Glans penis	2	0.1	Untransformed	0.2
Cowper's glands	2	0.1	Untransformed	0.2
Cowper's glands	8	0.1	Untransformed	0.2
Cowper's glands	14	0.1	Untransformed	0.2

LOEL, lowest observed effect level; SVCG, seminal vesicles and coagulating glands; LABC, levator ani and bulbo-cavernosus muscles; COWS, Cowper's glands; TP, testosterone propionate.

Suppl. Material Table 15. Coefficients of Variation for Body Weights and Male Accessory Tissues in Phase-1A

	Starting BW	Terminal BW	Ventral Prostate	SVCG	LABC	Glans Penis	Cowper's Glands
Lab 1	7.1%	6.0%	19.4%	14.0%	11.5%	7.9%	13.9%
Lab 2	4.2%	4.7%	27.6%	29.4%	18.4%	23.7%	29.0%
Lab 3	4.6%	5.5%	20.8%	19.5%	13.4%	16.3%	17.3%
Lab 4	5.7%	5.3%	24.8%	15.7%	23.4%	24.8%	31.2%
Lab 5	3.9%	4.3%	19.9%	22.6%	8.0%	8.6%	25.5%
Lab 6	4.4%	5.0%	29.4%	19.7%	12.1%	11.1%	17.6%
Lab 7	5.7%	5.7%	42.9%	28.5%	23.0%	18.3%	40.6%
Lab 8	3.4%	4.4%	18.5%	20.8%	8.4%	5.3%	23.0%
Lab 9	3.1%	4.7%	42.2%	36.1%	16.8%	11.9%	25.8%
Lab 10	3.6%	4.1%	20.7%	14.6%	9.0%	11.7%	13.2%
Lab 12	4.4%	5.1%	18.2%	18.7%	10.8%	11.7%	24.2%
Lab 13	2.7%	3.7%	19.8%	17.1%	10.3%	11.4%	19.1%
Lab 14	3.7%	3.3%	15.9%	20.6%	11.5%	11.3%	18.9%
Lab 15	4.4%	4.7%	17.0%	15.6%	9.8%	11.2%	23.0%
Lab 16	3.9%	5.1%	24.0%	13.9%	9.8%	10.0%	18.4%
Lab 17	4.0%	4.3%	17.5%	13.9%	10.5%	9.5%	21.2%
Average CV	4.30%	4.74%	23.66%	20.04%	12.92%	12.79%	22.62%

BW, body weight; SVCG, Seminal vesicles and coagulating glands; LABC, Levator ani and bulbocavernosus muscles

Suppl. Material Table 16. Coefficients of Variation for Body Weights and Male Accessory Tissues in Phase-1B

	TP Dose	Starting BW	Terminal BW	Ventral Prostate	SVCG	LABC	Glans Penis	Cowper's Glands
Lab 5	0.2	4.26%	4.47%	16.0%	18.1%	9.3%	7.0%	23.6%
	0.4	4.51%	5.42%	20.5%	17.7%	11.5%	6.1%	20.0%
Lab 8	0.2	3.43%	4.93%	34.2%	27.3%	11.8%	12.4%	22.5%
Lab 10	0.4	2.72%	3.11%	19.3%	16.7%	10.1%	10.7%	15.8%
Lab 12	0.2	4.14%	5.26%	20.8%	24.3%	13.1%	7.0%	16.5%
	0.4	4.33%	5.71%	24.4%	22.7%	12.2%	4.7%	15.1%
Lab 13	0.2	2.70%	3.77%	21.7%	20.5%	13.7%	10.3%	21.3%
	0.4	3.17%	4.59%	18.8%	22.2%	8.9%	9.3%	16.4%
Lab 15	0.2	4.07%	4.50%	16.2%	22.8%	11.0%	11.2%	21.1%
	0.4	3.85%	4.25%	15.1%	15.3%	9.8%	10.5%	17.8%
Lab 17	0.2	3.44%	4.51%	22.6%	22.6%	13.4%	13.8%	26.5%
Average CV		3.69%	4.59%	20.87%	20.93%	11.35%	9.36%	19.69%

BW, body weight; SVCG, Seminal vesicles and coagulating glands; LABC, Levator ani and bulbocavernosus muscles

Section IV. An example of the statistical output for one of the Dunnett's analyses.

The following is the statistical output for Laboratory in Phase 1B using the final, necropsy body weights. For each tissue there are a series of outputs, so that when the data are transformed (log 10) or adjusted for body weight using ANCOVA (bw) should be transparent. The final output are the Flutamide treatment groups compared against the TP stimulating dose, 0.4 mg TP/kg-bw/d treatment group in this study. The results are reported as the estimated mean (estimate) relative to the TP treated group, the lower 95% confidence level of the relative mean (lower) , and the upper 95% confidence level relative of the mean (upper). In these cases, when the upper 95% confidence level is < 1.000, then p< 0.05 and that group is statistically different.

Additionally, the Studentized residuals were plotted in the analysis for each tissue. One Figure is attached for this study that indicated that one individual data point in a group did have a Studentized residual indicated that it was an outlier (± 3.75).

VP Analysis:

Analysis of Variance Table
Response: log10(bw)
Terms added sequentially (first to last)

	Df	Sum of Sq	Mean Sq	F Value	Pr(F)
group	5	0.00152251	0.0003045030	0.7228465	0.6115571
Residuals	30	0.01263766	0.0004212554		

Analysis of Variance Table
Response: log10(vp)
Type III Sum of Squares

	Df	Sum of Sq	Mean Sq	F Value	Pr(F)
log10(bw)	1	0.026150	0.0261497	4.03775	0.05388056
group	5	2.305602	0.4611205	71.20117	0.00000000
Residuals	29	0.187813	0.0064763		

Analysis of Variance Table
Response: log10(vp)
Type III Sum of Squares

	Df	Sum of Sq	Mean Sq	F Value	Pr(F)
log10(bw)	1	0.0250160	0.02501595	3.594447	0.0700764
group	5	0.0219185	0.00438370	0.629877	0.6787156
log10(bw):group	5	0.0207822	0.00415643	0.597222	0.7023602
Residuals	24	0.1670307	0.00695961		

Analysis of Variance Table
Response: log10(vp)
Type III Sum of Squares

	Df	Sum of Sq	Mean Sq	F Value	Pr(F)
bw	1	0.026337	0.0263373	4.07079	0.05296898
group	5	2.296506	0.4593011	70.99116	0.00000000
Residuals	29	0.187625	0.0064698		

Analysis of Variance Table
Response: log10(vp)
Type III Sum of Squares

	Df	Sum of Sq	Mean Sq	F Value	Pr(F)
bw	1	0.0252502	0.02525024	3.626158	0.0689333
group	5	0.0302325	0.00604650	0.868330	0.5166676
bw:group	5	0.0205046	0.00410092	0.588929	0.7083906
Residuals	24	0.1671206	0.00696336		

	estimate	lower	upper
TP 0.4 + FLUT 0.1-0.4 TP only	0.9909698	0.7453480	1.3175337
TP 0.4 + FLUT 0.3-0.4 TP only	0.8700589	0.6540089	1.1574805
TP 0.4 + FLUT 1-0.4 TP only	0.7997598	0.5968557	1.0716423
TP 0.4 + FLUT 3-0.4 TP only	0.3598527	0.2691071	0.4811984
TP 0.4+ FLUT 10-0.4 TP only	0.2036909	0.1517950	0.2733290

SVCG Analysis:

Analysis of Variance Table
Response: log10(bw)
Terms added sequentially (first to last)

	Df	Sum of Sq	Mean Sq	F Value	Pr(F)
group	5	0.00152251	0.0003045030	0.7228465	0.6115571
Residuals	30	0.01263766	0.0004212554		

Analysis of Variance Table
Response: log10(svcg)
Type III Sum of Squares

	Df	Sum of Sq	Mean Sq	F Value	Pr(F)
log10(bw)	1	0.013378	0.0133777	1.13345	0.2958245
group	5	4.060546	0.8121093	68.80749	0.0000000
Residuals	29	0.342276	0.0118026		

Analysis of Variance Table
Response: log10(svcg)
Type III Sum of Squares

	Df	Sum of Sq	Mean Sq	F Value	Pr(F)
log10(bw)	1	0.0076274	0.00762743	0.6131637	0.4412580
group	5	0.0432230	0.00864460	0.6949332	0.6323424
log10(bw):group	5	0.0437289	0.00874579	0.7030675	0.6266343
Residuals	24	0.2985473	0.01243947		

Analysis of Variance Table
Response: log10(svcg)
Type III Sum of Squares

	Df	Sum of Sq	Mean Sq	F Value	Pr(F)
bw	1	0.013974	0.0139739	1.18603	0.2850989
group	5	4.055894	0.8111788	68.84858	0.0000000
Residuals	29	0.341680	0.0117821		

Analysis of Variance Table
Response: log10(svcg)
Type III Sum of Squares

	Df	Sum of Sq	Mean Sq	F Value	Pr(F)
bw	1	0.0083478	0.00834780	0.6736424	0.4198626
group	5	0.0465190	0.00930379	0.7507882	0.5936419
bw:group	5	0.0442712	0.00885424	0.7145108	0.6186438
Residuals	24	0.2974088	0.01239203		

	estimate	lower	upper
TP 0.4 + FLUT 0.1-0.4 TP only	0.9626697	0.65545927	1.4138682
TP 0.4 + FLUT 0.3-0.4 TP only	0.8391032	0.57085782	1.2333968
TP 0.4 + FLUT 1-0.4 TP only	0.5719432	0.38534348	0.8489023
TP 0.4 + FLUT 3-0.4 TP only	0.2304033	0.15566319	0.3410292
TP 0.4+ FLUT 10-0.4 TP only	0.1184759	0.07966789	0.1761882

LABC Analysis:

Analysis of Variance Table
Response: log10(bw)
Terms added sequentially (first to last)

	Df	Sum of Sq	Mean Sq	F Value	Pr(F)
group	5	0.00152251	0.0003045030	0.7228465	0.6115571
Residuals	30	0.01263766	0.0004212554		

Analysis of Variance Table
Response: log10(labc)
Type III Sum of Squares

	Df	Sum of Sq	Mean Sq	F Value	Pr(F)
log10(bw)	1	0.0010419	0.0010419	0.58578	0.4502392
group	5	0.6439261	0.1287852	72.40445	0.0000000
Residuals	29	0.0515821	0.0017787		

Analysis of Variance Table
Response: log10(labc)
Type III Sum of Squares

	Df	Sum of Sq	Mean Sq	F Value	Pr(F)
log10(bw)	1	0.00151887	0.001518874	0.8007523	0.3797556
group	5	0.00568968	0.001137936	0.5999213	0.7003994
log10(bw):group	5	0.00605866	0.001211732	0.6388267	0.6722703
Residuals	24	0.04552341	0.001896809		

Analysis of Variance Table
Response: log10(labc)
Type III Sum of Squares

	Df	Sum of Sq	Mean Sq	F Value	Pr(F)
bw	1	0.0010383	0.0010383	0.58371	0.4510322
group	5	0.6421171	0.1284234	72.19599	0.0000000
Residuals	29	0.0515857	0.0017788		

Analysis of Variance Table
Response: log10(labc)
Type III Sum of Squares

	Df	Sum of Sq	Mean Sq	F Value	Pr(F)
bw	1	0.00146473	0.001464731	0.7704333	0.3887856
group	5	0.00456796	0.000913592	0.4805400	0.7872072
bw:group	5	0.00595741	0.001191482	0 .6267074	0.6810020
Residuals	24	0.04562827	0.001901178		

	estimate	lower	upper
TP 0.4 + FLUT 0.1-0.4 TP only	0.9074737	0.7815767	1.0536504
TP 0.4 + FLUT 0.3-0.4 TP only	0.8470285	0.7292851	0.9837817
TP 0.4 + FLUT 1-0.4 TP only	0.7457112	0.6396335	0.8693808
TP 0.4 + FLUT 3-0.4 TP only	0.5025897	0.4315600	0.5853099
TP 0.4+ FLUT 10-0.4 TP only	0.4165134	0.3569953	0.4859544

GP Analysis:

Analysis of Variance Table
Response: log10(bw)
Terms added sequentially (first to last)

	Df	Sum of Sq	Mean Sq	F Value	Pr(F)
group	5	0.00152251	0.0003045030	0.7228465	0.6115571
Residuals	30	0.01263766	0.0004212554		

Analysis of Variance Table
Response: log10(gp)
Type III Sum of Squares

	Df	Sum of Sq	Mean Sq	F Value	Pr(F)
log10(bw)	1	0.0012656	0.00126557	0.75468	0.3921303
group	5	0.2162822	0.04325645	25.79459	0.0000000
Residuals	29	0.0486318	0.00167696		

Analysis of Variance Table
Response: log10(gp)
Type III Sum of Squares

	Df	Sum of Sq	Mean Sq	F Value	Pr(F)
log10(bw)	1	0.00093287	0.000932869	0.5328871	0.4724613
group	5	0.00639069	0.001278138	0.7301170	0.6078246
log10(bw):group	5	0.00661756	0.001323511	0.7560359	0.5900694
Residuals	24	0.04201423	0.001750593		

Analysis of Variance Table
Response: log10(gp)
Type III Sum of Squares

	Df	Sum of Sq	Mean Sq	F Value	Pr(F)
bw	1	0.0013417	0.00134172	0.80135	0.3780609
group	5	0.2153606	0.04307212	25.72495	0.0000000
Residuals	29	0.0485556	0.00167433		

EHP 8751: OECD Validation of Rat Hershberger Assay: Phase-1

Analysis of Variance Table
Response: log10(gp)
Type III Sum of Squares

	Df	Sum of Sq	Mean Sq	F Value	Pr(F)
bw	1	0.00093215	0.000932147	0.5325378	0.4726049
group	5	0.00545323	0.001090646	0.6230890	0.6836149
bw:group	5	0.00654637	0.001309274	0.7479914	0.5955505
Residuals	24	0.04200927	0.001750386		

	estimate	lower	upper
TP 0.4 + FLUT 0.1-0.4 TP only	0.9786780	0.8466641	1.1312758
TP 0.4 + FLUT 0.3-0.4 TP only	0.9292471	0.8036527	1.0744694
TP 0.4 + FLUT 1-0.4 TP only	0.8723077	0.7516523	1.0123306
TP 0.4 + FLUT 3-0.4 TP only	0.7113007	0.6135553	0.8246180
TP 0.4+ FLUT 10-0.4 TP only	0.6008260	0.5173431	0.6977803

COWS Analysis:

Analysis of Variance Table
Response: log10(bw)
Terms added sequentially (first to last)

	Df	Sum of Sq	Mean Sq	F Value	Pr(F)
group	5	0.00152251	0.0003045030	0.7228465	0.6115571
Residuals	30	0.01263766	0.0004212554		

Analysis of Variance Table
Response: log10(cows)
Type III Sum of Squares

	Df	Sum of Sq	Mean Sq	F Value	Pr(F)
log10(bw)	1	0.001732	0.0017318	0.31919	0.5764388
group	5	1.184167	0.2368333	43.65046	0.0000000
Residuals	29	0.157345	0.0054257		

Analysis of Variance Table
Response: log10(cows)
Type III Sum of Squares

	Df	Sum of Sq	Mean Sq	F Value	Pr(F)
log10(bw)	1	0.0006951	0.000695109	0.131926	0.7196227
group	5	0.0311158	0.006223154	1.181106	0.3475193
log10(bw):group	5	0.0308905	0.006178108	1.172557	0.3514344
Residuals	24	0.1264541	0.005268921		

Analysis of Variance Table
Response: log10(cows)
Type III Sum of Squares

	Df	Sum of Sq	Mean Sq	F Value	Pr(F)
bw	1	0.001682	0.0016823	0.30997	0.5819706
group	5	1.181340	0.2362681	43.53258	0.0000000
Residuals	29	0.157394	0.0054274		

EHP 8751: OECD Validation of Rat Hershberger Assay: Phase-1

Analysis of Variance Table
Response: log10(cows)
Type III Sum of Squares

	Df	Sum of Sq	Mean Sq	F Value	Pr(F)
bw	1	0.0005628	0.000562820	0.107323	0.7460533
group	5	0.0343567	0.006871346	1.310284	0.2929229
bw:group	5	0.0315342	0.006306839	1.202639	0.3378282
Residuals	24	0.1258600	0.005244165		

	estimate	lower	upper
TP 0.4 + FLUT 0.1-0.4 TP only	1.0154218	0.7822548	1.3180889
TP 0.4 + FLUT 0.3-0.4 TP only	0.8091959	0.6230372	1.0509774
TP 0.4 + FLUT 1-0.4 TP only	0.7501726	0.5737981	0.9807613
TP 0.4 + FLUT 3-0.4 TP only	0.4819176	0.3693061	0.6288673
TP 0.4+ FLUT 10-0.4 TP only	0.3026185	0.2311650	0.3961585

The Figure is a plot of the Studentized residuals for the vental prostate including both the original untransformed data on the left and after log transformation on the right. Note that there was an outlier before log transformation of these data.

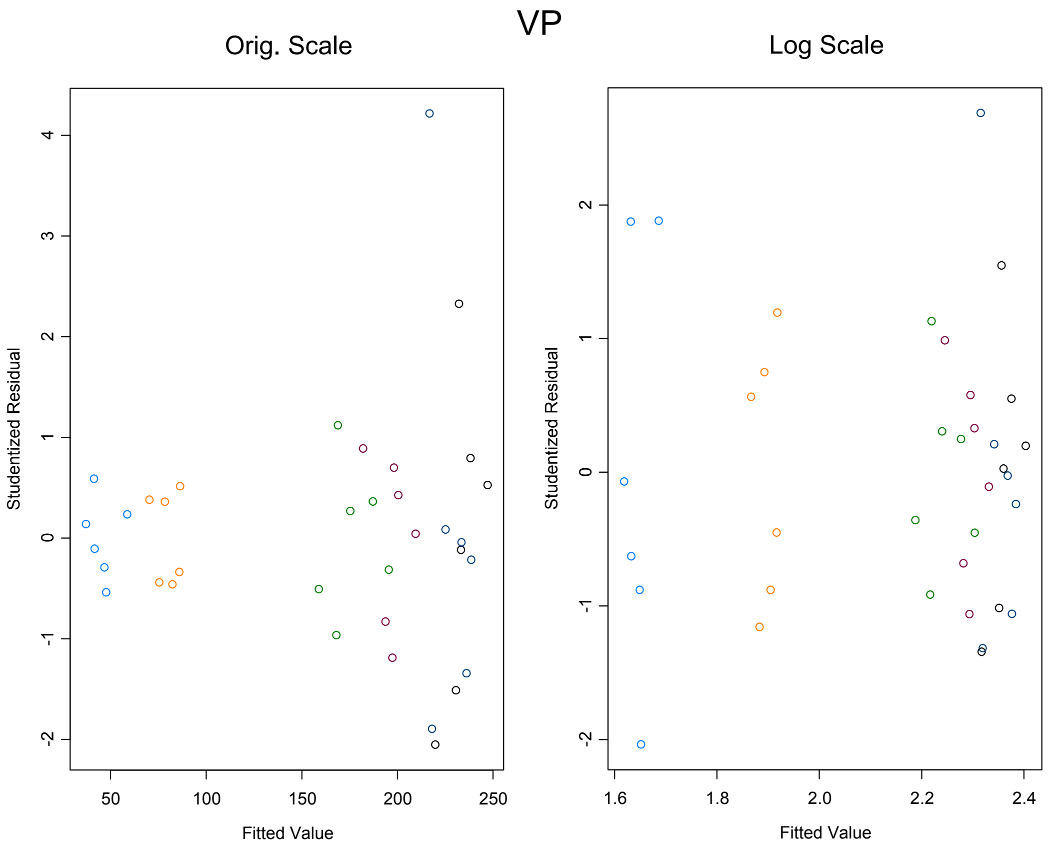

Supplement: Supplemental Figures and Tables [file ehp0114-001259s1.pdf]
